# Sensitivity Enhancement by Progressive Saturation of the Proton Reservoir: A Solid-State NMR Analogue of Chemical Exchange Saturation Transfer

**DOI:** 10.1021/jacs.1c08277

**Published:** 2021-11-18

**Authors:** Michael J. Jaroszewicz, Adam R. Altenhof, Robert W. Schurko, Lucio Frydman

**Affiliations:** † Department of Chemical and Biological Physics, Weizmann Institute of Science, Rehovot 7610001, Israel; ‡ Department of Chemistry and Biochemistry, Florida State University, Tallahassee, Florida 32306, United States; § National High Magnetic Field Laboratory, 1800 East Paul Dirac Drive, Tallahassee, Florida 32310, United States

## Abstract

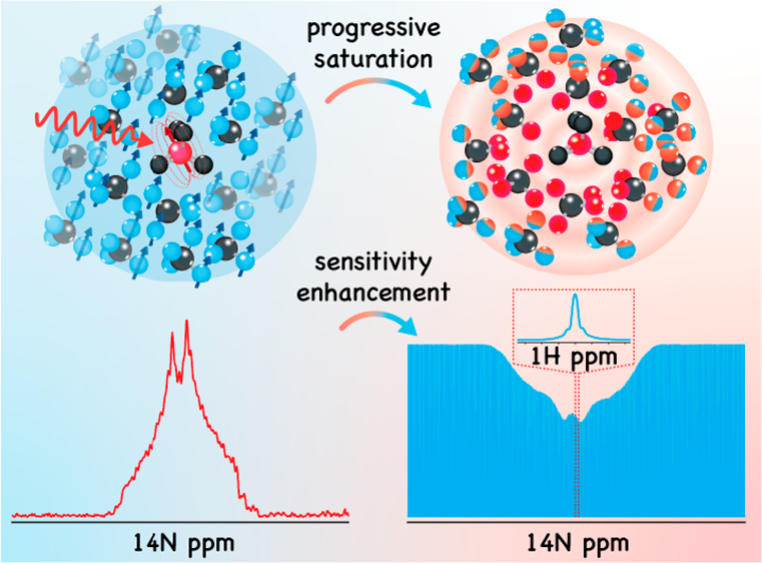

Chemical exchange saturation transfer (CEST) enhances solution-state NMR signals of labile and otherwise invisible chemical sites, by indirectly detecting their signatures as a highly magnified saturation of an abundant resonance—for instance, the ^1^H resonance of water. Stimulated by this sensitivity magnification, this study presents PROgressive Saturation of the Proton Reservoir (PROSPR), a method for enhancing the NMR sensitivity of dilute heteronuclei in static solids. PROSPR aims at using these heteronuclei to progressively deplete the abundant ^1^H polarization found in most organic and several inorganic solids, and implements this ^1^H signal depletion in a manner that reflects the spectral intensities of the heteronuclei as a function of their chemical shifts or quadrupolar offsets. To achieve this, PROSPR uses a looped cross-polarization scheme that repeatedly depletes ^1^H–^1^H local dipolar order and then relays this saturation throughout the full ^1^H reservoir via spin-diffusion processes that act as analogues of chemical exchanges in the CEST experiment. Repeating this cross-polarization/spin-diffusion procedure multiple times results in an effective magnification of each heteronucleus’s response that, when repeated in a frequency-stepped fashion, indirectly maps their NMR spectrum as sizable attenuations of the abundant ^1^H NMR signal. Experimental PROSPR examples demonstrate that, in this fashion, faithful wideline NMR spectra can be obtained. These ^1^H-detected heteronuclear NMR spectra can have their sensitivity enhanced by orders of magnitude in comparison to optimized direct-detect experiments targeting unreceptive nuclei at low natural abundance, using modest hardware requirements and conventional NMR equipment at room temperature.

## Introduction

Chemical exchange saturation transfer (CEST)^1^ is an approach that can dramatically enhance the NMR sensitivity of nuclei in sites undergoing chemical exchange and has opened up numerous applications in solution-state magnetic resonance.^2−6^ CEST amplifies the signatures of labile protons in metabolites and biomacromolecules by up to 3 orders of magnitude, leading to unprecedented imaging possibilities and providing a unique source of metabolic *in vivo* MRI contrast.^7−12^ CEST also enables the detection of invisible structural conformation states in the solution NMR of nucleic acids^13,14^ and proteins,^15−18^ the observation of transient reaction intermediates,^19^ and the enhancement of structurally relevant cross peaks in multidimensional correlation NMR measurements.^20,21^ CEST magnifies the weak response from dilute chemical sites by repeatedly saturating their spin polarization using a weak radio frequency (RF) field and then relying on chemical exchanges to pass this information to an abundant spin pool with a much stronger NMR resonance, which can report on the dilute spins’ spectrum with a substantial sensitivity enhancement. Given a chemical exchange rate *k*_ex_ between the dilute and abundant spin pools and a longitudinal relaxation time *T*_1_ for the abundant spins, this signal magnification can be on the order of *k*_ex_ × *T*_1_. This factor can exceed the original signal from the dilute spins by several orders of magnitude, providing dramatic increases in sensitivity from on/off saturation-difference experiments. Besides providing an on/off contrast, this concept also allows for the indirect detection of the dilute spins’ NMR spectrum, by stepping the carrier frequency of the saturation pulse over a suitable spectral range. Measuring and plotting the resulting drop in the resonance of the abundant spins as a function of the RF offset provides a so-called *z* spectrum of the labile sites,^22,23^ which is akin to a normal NMR spectrum, but with a dramatically enhanced signal-to-noise ratio (SNR). This leveraging of saturation and chemical exchanges to indirectly map the NMR spectra of dilute sites has led to the generation of numerous solution-state NMR methods that provide SNR enhancements not only for labile sites^24^ but also for nonlabile^25^ and heteronuclear ones.^26−28^ Sites of interest that give rise to weak signals as a result of chemical dilution or low natural abundance, or both of these, can thus have their NMR spectra magnified (under suitable conditions) by factors comparable to those obtained by nuclear hyperpolarization.

These CEST ideas and experiments have found extensive uses in solution-state NMR. Even when targeting semisolid tissues *in vivo*, it is chemical exchange with water molecules and their ∼50 M ^1^H pool that ends up providing the desired signal enhancement. By contrast, the present study introduces and exemplifies a saturation transfer-based method designed to specifically enhance the NMR sensitivity of dilute heteronuclei in polycrystalline solids. Unlike in its solution-state counterparts, a water resonance involving protons that can undergo chemical exchanges with a labile site of interest is not available. As an alternative, the NMR experiment here envisioned relies on the highly abundant pool of protons that is typically present in polycrystalline solids. This is a polarization reservoir that is usually underutilized in heteronuclear-based NMR experiments (*vide infra*). It is via the depletion of this abundant ^1^H signal that we propose to magnify the NMR signals of dilute heteronuclei, in what we denominate the PROgressive Saturation of the Proton Reservoir (PROSPR) approach for recording static solid-state NMR spectra. The details pertaining to the engineering of such a method functioning as a solid-state NMR analogue of CEST are discussed in the following section.

## Method

**Figure 1:**
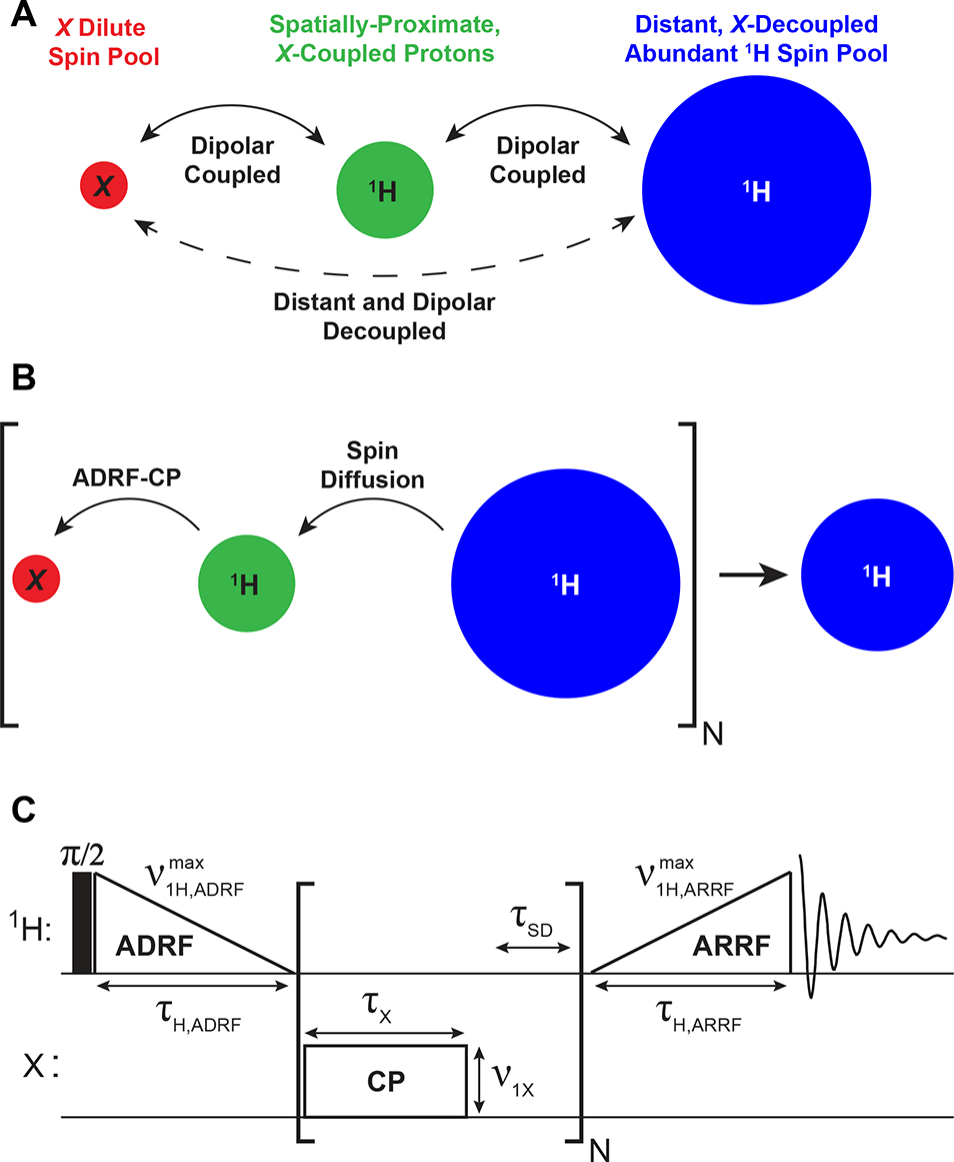
(A) Illustration showing how a typical organic solid can be classified into three spin pools on the basis of heteronuclear dipolar couplings, where the size of each circle indicates the relative polarization of each pool. (B) Conceptual principle of the PROSPR experiment, whereby repeated green → red cross-polarization and blue → green spin-diffusion processes end up depolarizing the abundant, ^1^H-reporting reservoir. (C) Schematic representation of the PROSPR pulse sequence, which is capable of implementing the scheme in part B and which was employed in this study.

The prototypical polycrystalline solid considered for the execution of PROSPR is here visualized as made up of three distinct spin pools (Figure 1A). These include a heteronuclear site whose signals we are interested in magnifying (red circle, *X*) and which, by virtue of its low chemical or isotopic natural abundance, is dilute in the sample. These *X* nuclei will couple, via the heteronuclear dipolar interaction, with a fraction of the total protons in the sample (green circle, heteronuclear dipolar-coupled ^1^H spins) to which they are proximate. These ^1^Hs are also dilute but are coupled via the homonuclear dipolar interaction to the majority of ^1^Hs in the sample (blue circle, abundant ^1^H spin pool). We identify this abundant reservoir of *X*-decoupled ^1^H spins in the naturally abundant solid as akin to CEST’s water ^1^H spin pool, and it is PROSPR’s aim to exploit it for facilitating the observation of the heteronuclear NMR spectrum. Unlike the solution-state ^1^H NMR case, however, the abundant and dilute ^1^H spin reservoirs cannot be differentiated on the basis of chemical shifts. Instead, we assume that the dilute ^1^H reservoir can be selectively targeted by exploiting differences among the ^1^H–*X* dipolar couplings exhibited by the protons in the “green” and “blue” pools. Moreover, we will rely on ^1^H–^1^H spin diffusion as the “exchange” mechanism for transferring polarization between the distinct ^1^H reservoirs. Provided that the rate of this ^1^H–^1^H spin diffusion—akin to the aforementioned *k*_ex_—is larger than the longitudinal relaxation rate (1/*T*_1_) of the abundant ^1^Hs, these should be able to report on *X*’s NMR spectrum with a substantial SNR amplification.

The overall multistep approach we propose for realizing this solid-state, CEST-like NMR experiment is presented in Figure 1B. The method begins with an RF module that transfers polarization from the strongly *X*-coupled ^1^Hs (green) to the heteronuclei (red), under conditions that depend on the chemical shift or quadrupolar offset of the *X* nuclei. This offset-dependent ^1^H → *X* cross-polarization (CP)^29−31^ makes an imprint of the heteronuclear NMR spectrum, fulfilling a role similar to that of the narrowband RF saturation for revealing the spectrum of the labile ^1^Hs in water-detected CEST. As a result of this selective CP, the *X*-coupled ^1^H polarization is partially depleted. Then, in an ensuing RF-free period τ_SD_ (Figure 1C), two concurrent events happen: The *X*-spin polarization generated from CP decays (e.g., by *T*_2_, inhomogeneous broadening, dipolar couplings, etc.), while spin diffusion (SD) mediates a transfer of polarization between the abundant (blue) and dilute (green) ^1^H pools. As a result of this, the “green” reservoir is repolarized to approximately its initial level, while the “blue” spins have their polarization slightly depleted. Repeating this CP/SD module multiple times magnifies the *X-*dependent saturation throughout the abundant ^1^H reservoir. Detection of the abundant ^1^H polarization with a simple excite/acquire module (and subtracting the data from a similar experiment but lacking the *X*-channel pulses) should reflect the NMR response of the *X* spins, with a potential sensitivity enhancement. It is important to highlight that experiments measuring heteronuclear spectra based on stepping the *X*-channel carrier frequency and measuring the resulting changes to the ^1^H signal have been reported in the solid state.^32−35^ The fundamental difference between those experiments and PROSPR is that the latter seeks to deplete the sample’s *entire*^1^H spin reservoir in every scan, rather than probing solely the changes imparted on directly coupled protons (which represent a smaller fraction of the total ^1^H signal).

A simple route to implement the looped CP/SD module needed by this procedure involves repeating a flipped-back Hartmann–Hahn CP contact^36−38^ that excites, spin-locks, and then stores back along the longitudinal axis all the nontransferred ^1^H polarization. This approach, however, poses a challenge: since these experiments are performed under stationary conditions on a homogeneously dipolar-broadened ^1^H resonance, the multiple ^1^H excite/spin-lock/store elements to be looped may lead to substantial ^1^H signal intensity losses. These losses could be avoided by using high (>100 kHz) spin-locking RF fields; however, matching such high fields on low-γ *X* nuclei is not straightforward hardware-wise, and the expectation of making such CP processes *X-*offset dependent, and thus capable of sampling a spectrum in a frequency-stepped manner, dwindles under such high-power conditions. Supplement 2 of the Supporting Information describes this phenomenon in greater detail. As an alternative, the dipolar-ordered-based^29,39−42^ pulse sequence in Figure 1C was tested. Here, an initial π/2 pulse excites the equilibrium magnetization of the entire ^1^H spin system, which is then subjected to an adiabatic demagnetization in the rotating frame (ADRF) using a linear RF ramp-down.^42^ At the end of this ADRF, the ^1^H spin system exists, for a duration on the order of its dipolar relaxation time constant, *T*_1D_, as a dipolar-ordered state; *X* spin-polarization can be drawn from this state using an RF pulse applied only on the *X*-channel, matching in its nutation frequency the average strength of the ^1^H–^1^H dipolar couplings. As the ^1^H NMR spectrum is homogeneously dipole-broadened, these ^1^H → *X* polarization transfers can be executed over a range of RF field strengths, including by relatively weak (∼5–10 kHz), offset-selective *X*-channel pulses. This makes the CP-based pulse sequence of Figure 1C well-suited both to multiple repeats as well as to the achievement of *X* spectral selectivity. This strategy was introduced into the overall depolarizing module shown within the brackets in Figure 1C, which loops *N* times the *X* CP pulse with an RF-free SD period, where the transverse *X* polarization dephases, while the *X*-coupled ^1^Hs are repolarized by the abundant ^1^H reservoir. Finally, in order to evaluate how much this looped CP/SD process has depleted the dipolar order from the entire ^1^H system, an adiabatic remagnetization in the rotating frame (ARRF) ^1^H pulse is applied, which converts the partially saturated dipolar order of this abundant reservoir into detectable transverse magnetization. Assuming that the ^1^H–^1^H dipolar order is depleted in a manner that is in proportion to the *X* signal intensity at a given transmitter offset, a replica of *X*’s wide-line NMR spectrum can be mapped out by stepping the *X*-channel RF carrier across a spectral range and monitoring the differentially attenuated ^1^H resonance.

Numerous assumptions—including issues concerning the selectivity of ADRF/ARRF cross-polarization, the proportionality between the *X* powder line shape intensity and the extent of ^1^H spin depolarization, and the capability of ^1^H–^1^H dipolar order to support saturation transfer experiments—underlie this scheme. These assumptions have to hold in order to obtain the desired facsimile of *X*’s NMR spectrum, enhanced in terms of its SNR per square root unit acquisition time (ε_√T_ = SNR/(√time)). In order to corroborate some of these assumptions, spin dynamics simulations were carried out (Supplements 3 and 4 in the SI), using both semiclassical and quantum mechanical approaches (the latter on a nine-spin system under stationary conditions). These simulations confirmed that ^1^H–^1^H dipolar order can indeed be depleted via heteronuclear CP and that this local dipolar order depletion can be replenished via spin diffusion from a remote reservoir, in a manner akin to what happens with longitudinal polarization. Furthermore, simulations show that the degree by which the bulk ^1^H–^1^H dipolar order will be depleted depends on *X*’s spectral intensity at a given RF offset and that the frequency bandwidth of this depletion at a given *X* offset will be on the order of the RF strength used in the CP. They also show that looping the CP/SD process can lead to an increased depletion of the bulk ^1^H signal, up to a maximum given by ca. half the total number of ^1^Hs in the abundant spin pool. Taken in unison, all of this bodes well for enabling the acquisition of accurately rasterized *X* NMR powder patterns, by monitoring their depleting effects on an abundant ^1^H resonance.

## Results and Discussion

The feasibility of using such principles to indirectly measure heteronuclear solid-state NMR spectra with enhanced SNR was experimentally explored. All experiments were performed on naturally abundant, as-received polycrystalline samples, packed into 4 mm glass NMR tubes; these were then inserted into a modified Chemagnetics HXY probe devoid of ^1^H background signal. All data sets were recorded at a field strength of *B*_0_ = 14.1 T with the sample temperature regulated at 20 °C. Additional experimental details are provided in Supplement 1 in the SI.

**Figure 2:**
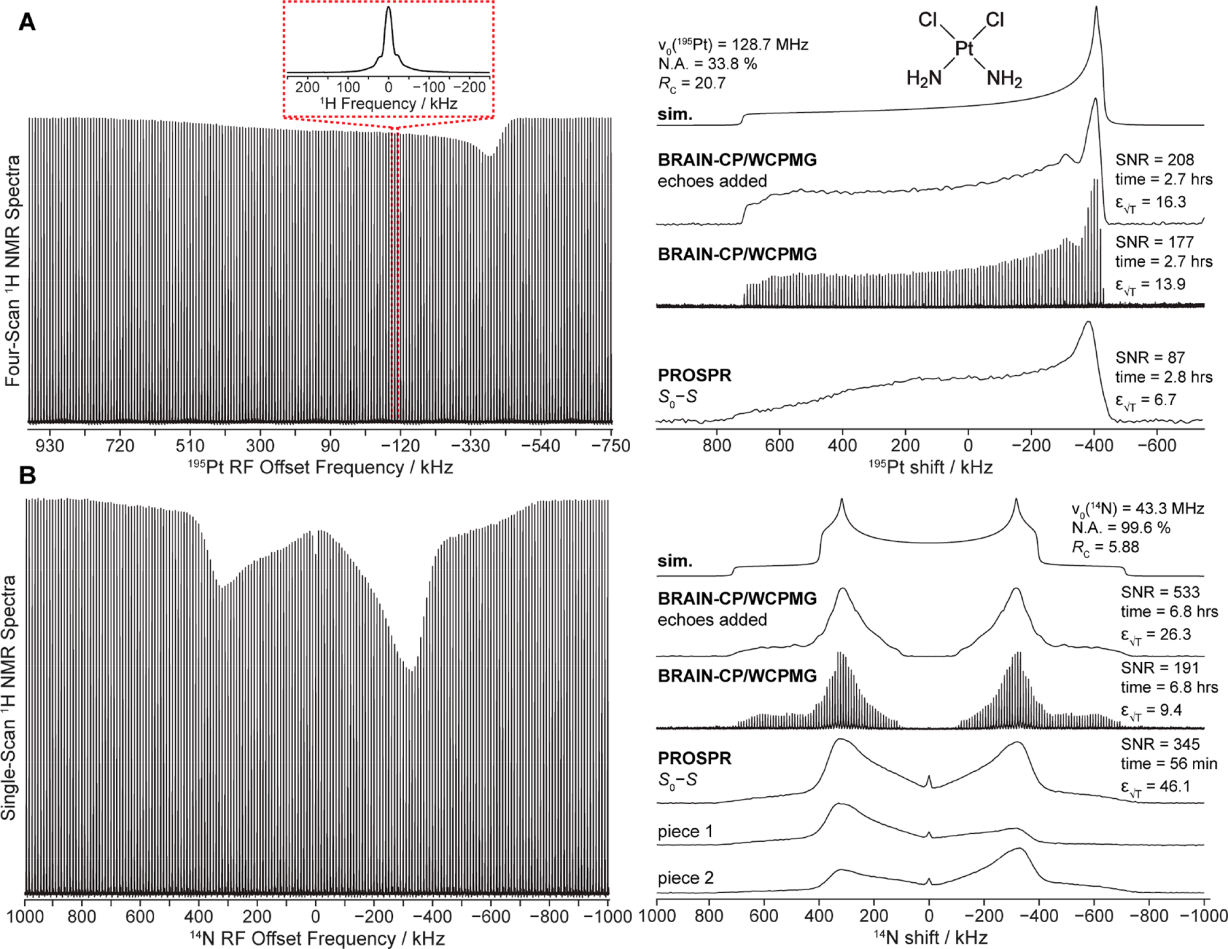
(A) ^195^Pt and (B) ^14^N NMR spectra collected with the PROSPR and WCPMG pulse sequences. The ^195^Pt and ^14^N *z* spectra are shown in the left column where each vertical line corresponds to a ^1^H NMR spectrum (shown in the inset), and the right column shows the *CP*(*off*) – *CP*(*on*) (*S*_0_ – *S*) postprocessed PROSPR spectra plotted against the optimized BRAIN-CP/WCPMG comparisons. ^195^Pt PROSPR spectra were collected with the *X*-channel tuned to a single frequency (ca. −380 kHz offset), whereas ^14^N PROSPR spectra were collected with the *X*-channel tuned to either one of the horn discontinuities (ca. ±350 kHz offsets); their coaddition is also shown as a 1D trace. A total of 7 and 8 subspectra were collected for the ^195^Pt and ^14^N BRAIN-CP/WCPMG spectra, respectively. Sensitivity values ε_√T_ reported as SNR/ are included, as are the SNR and acquisition times of each experiment. Additional experimental details are contained within Supplement 1 and Tables S2–S5. Larmor frequencies, natural abundances (N.A.), and receptivities with respect to ^13^C (*R*_C_) are provided in the figure.

Figure 2 shows PROSPR NMR *z* spectra collected for a sample of cisplatin, contrasted with those acquired under optimized conditions with BRAIN-CP/WCPMG^43,44^—a pulse sequence optimized for acquiring high-quality wide-line NMR spectra under static conditions. Figure 2A shows the *z* spectrum collected using Figure 1C for ^195^Pt, a relatively abundant spin-1/2 nucleus whose powder pattern is dominated by chemical shift anisotropy and spans ca. 1.4 MHz at this field. Figure 2B shows results obtained for the ^14^N in the sample, a spin-1 nucleus whose first-order quadrupolar powder pattern spans nearly 2 MHz. Both cases demonstrate PROSPR’s ability to encode and detect an *X* NMR spectrum by measuring the attenuated ^1^H signals, arising after repeatedly depolarizing the dipole-ordered ^1^H reservoir. The maximal saturation imparted on the ^1^H NMR signal in these variable offset experiments was ca. 30% and 10% for ^14^N and ^195^Pt, respectively. These were achieved using a ^14^N/^195^Pt RF field strength of ca. 10 kHz with a contact time of 15 ms and *N* = 60/65 loops (Figures S4 and S5), values that are consistent with an RF-driven matching to local dipolar ^1^H fields lasting for a *T*_1D_ ≈ 2 s (Figure S2). When compared against a literature-based simulation^45^ and an optimized BRAIN-CP/WCPMG NMR spectrum, the ^195^Pt PROSPR trace that arises upon subtracting the depolarized ^1^H spectrum *S* from a reference ^1^H spectrum *S*_0_ lacking the ^195^Pt pulses reveals a comparable line shape and similar SNR per unit time—even though the PROSPR data was acquired with the *X* channel tuned to a single offset frequency, while BRAIN-CP/WCPMG required 7 different ^195^Pt offsets in its collection. For the more challenging ^14^N powder pattern example, however, PROSPR already reveals a significant  enhancement in comparison to its BRAIN-CP/WCPMG optimized counterpart, having comparable SNR (345 vs 533, respectively) but requiring much shorter acquisition times (56 min vs 6.8 h, respectively). Furthermore, whereas BRAIN-CP/WCPMG required 8 different ^14^N offsets to deliver a line shape that still evidence distortions (e.g., close to the zero-frequency region), PROSPR only required 2 subspectra to reconstruct the total ^14^N pattern (Figure 2B, right). This reflects the additional robustness endowed by looped experiments, which tend to drive the CP spin dynamics toward thermodynamic completion despite kinetics limitations imposed by Hartmann–Hahn mismatches or electronics-related bandwidth restraints.^24^ It also reflects the fact that, rather than matching a particular resonant condition, ADRF-based CP seeks to match a dipole-broadened frequency spectrum.

**Figure 3:**
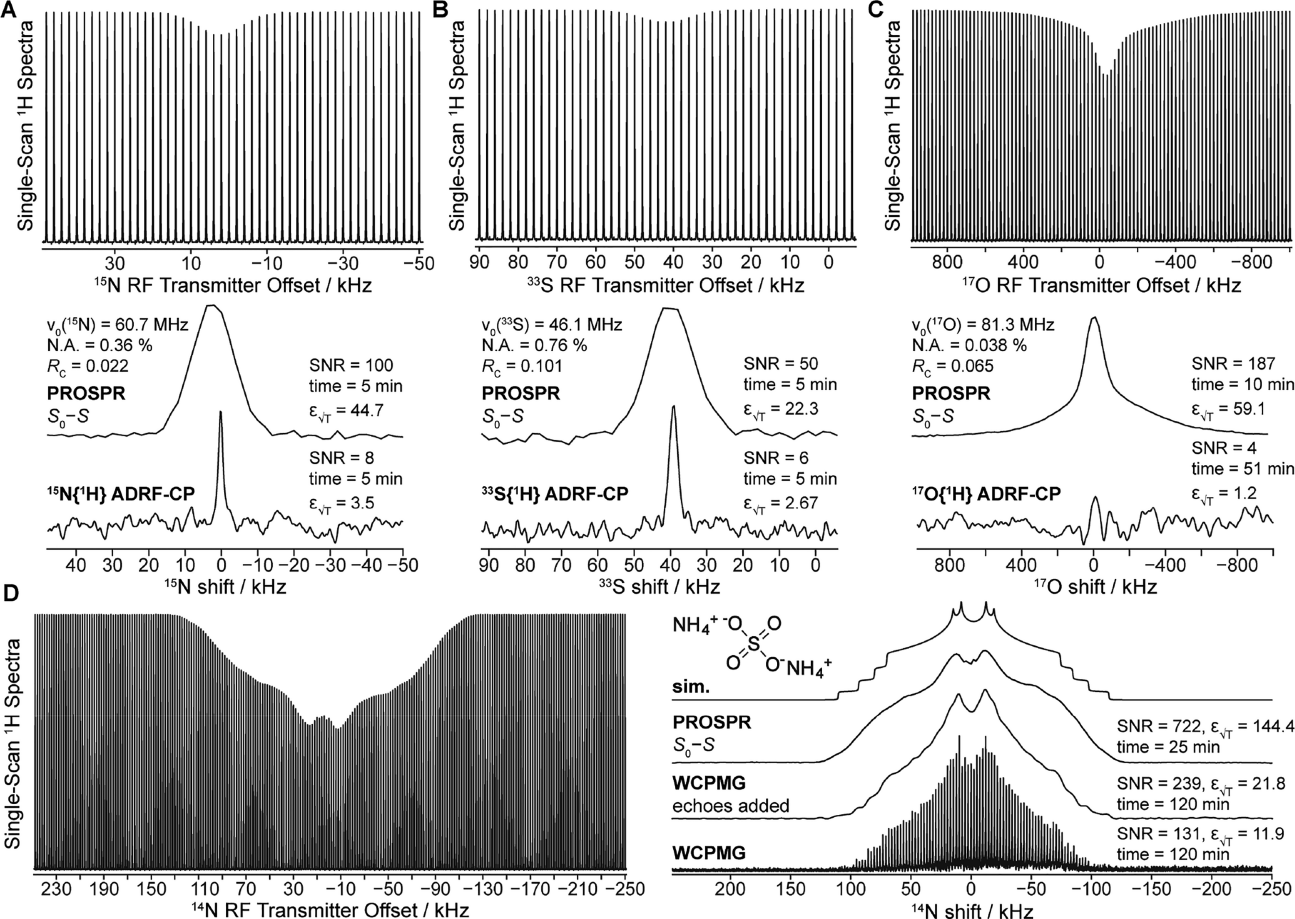
(A) ^15^N, (B) ^33^S, and (C) ^17^O NMR spectra collected with the PROSPR and ADRF-CP pulse sequences. (D) ^14^N NMR spectra collected with the PROSPR and BRAIN-CP/WCPMG pulse sequences. All spectra were acquired on the same ammonium sulfate sample with the *X*-channel tuned each time to a single fixed frequency. Symbols are as in Figure 2; additional experimental details are contained within Supplement 1 and Tables S6–S13.

Figure 3 presents additional examples targeting some of the challenging nuclides present in a sample of ammonium sulfate. PROSPR NMR spectra are shown for three nuclides, present in this powder at low natural abundances: ^15^N (spin-1/2, Figure 3A), ^33^S (spin-3/2, Figure 3B), and ^17^O (spin-5/2, Figure 3C). Compared against these are conventional NMR spectra collected for the same sample under optimized ADRF/ARRF CP conditions.^29,46^ These single-site spectra clearly highlight the significant SNR enhancements that can be achieved by PROSPR: ε_√T_ = 12.7, 8.5, and >49.2 min^–1/2^, for the ^15^N, ^33^S, and ^17^O cases, respectively. For the ^17^O PROSPR case, there is no clear second-order line shape at this field, yet an isotropic-like shift of 215.4 ppm (vs H_2_^17^O) is evidenced; notice as well the emergence of a broader line shape, presumably associated with the satellite transitions. For completion, Figure 3D presents an ^14^N example for the same sample, demonstrating PROSPR’s ability to reveal the two ca. 250 kHz-wide overlapping ^14^N patterns, while providing higher SNR in a shorter acquisition time vs an optimized WCPMG comparison.^47,48^

It is important to highlight the differences in the powder pattern line shapes arising from the directly detected vs the PROSPR experiments: in all cases, PROSPR patterns appear to be convolved with broader point-spread functions (Figures 2 and 3). This increased line width results from the limited spectral selectivity of the cross-polarizing *X* RF pulse used to impart PROSPR’s offset-dependent depletion; this is akin to so-called spillover effects usually broadening line shapes in CEST-based *z* spectra. For PROSPR NMR spectra, further broadenings are expected from the absence of active ^1^H heteronuclear decoupling during the encoding of the heteronuclear NMR spectrum. These effects and their implications for measuring accurate NMR line shapes are further analyzed in Supplements 4 and 5 in the SI.

## Conclusions

These results illustrate the potential for using CEST-inspired concepts for enhancing the solid-state NMR detectability of unreceptive, dilute heteronuclei. Results targeting more abundant ^14^N and ^195^Pt species evidenced the quality of the achievable line shapes, while targeting dilute species like ^33^S or ^17^O showed the experiment’s signal enhancement potential when applied to dilute species. As in CEST, PROSPR achieves this enhancement by exploiting spin polarization that would otherwise go unused. As opposed to monitoring an abundant solvent upon saturating labile sites undergoing chemical exchanges, this solid-state NMR analogue relies on progressively saturating an abundant ^1^H reservoir via repeated CP/SD events. The ensuing degree of signal enhancement will depend on the ratio between the abundance of ^1^Hs in the sample vs the concentration of the targeted nuclei: the larger this ratio, the bigger the potential method’s enhancement. Practical considerations led us to explore the use of ^1^H–^1^H dipolar order instead of ^1^H Zeeman order for performing the looped procedures underlying the depolarization of the bulk ^1^H reservoir. The conditions needed for performing PROSPR then ended up being quite favorable (Supporting Information, Figures S4–S9): these included low RF powers achievable by any solid-state (and most solution-state) NMR setup, compatibility with the application of numerous (*N* ≥ 70) depolarizing loops leading to low duty cycles, the achievement of a relatively narrow point-spread function capable of defining the *X* NMR spectrum with accuracy, an ability to record wide-line NMR patterns without having to retune the *X* channel across the breadth of the targeted powder pattern, and a minimization of RF-based manipulations that executed the ^1^H reservoir (whose presence would lead to an increase in artifacts and *t*_1_-like noise). At the same time, dipolar order-based approaches have evident drawbacks, including the need for long *T*_1D_ times (something that is not always under control of the experimentalist), and incompatibilities with both magic-angle spinning and heteronuclear decoupling—even if the generation of a certain amount of dipolar order has also been observed for rotating solids.^49^ Furthermore, not all materials—particularly not all inorganic systems—have an abundant pool of ^1^Hs that can act as a reporter. An upcoming study will also illustrate how some of these drawbacks can be ameliorated and in turn lead to higher-resolution solid-state MAS NMR spectra benefiting from CEST-like sensitivity enhancements. Still, even in its current, limited-resolution format, one can envision PROSPR as having valuable applications for wide-line NMR acquisitions of nuclei that—because of either isotopic composition or chemical dilution—have a low abundance in the sample.

## Supporting Information Available

Experimental Details and Optimization of PROSPR Experiments: samples, calibrations, data processing, optimization; Decay of spin-locked ^1^H polarizations upon looping the cross-polarization spin-lock; Numerical Simulations of PROSPR’s Dipolar-Order Spin Dynamics; PROSPR as a Two-Site Exchange Using the Bloch-McConnell Formalism: Basic Features of PROSPR’s Point-Spread Function; Comparing the Sensitivity and Spectral Resolution Afforded by ADRF-CP, PROSPR, and CP/MAS for ^15^N NMR Spectroscopy of Ammonium Sulfate (PDF)

## Abbreviations

ADRF: adiabatic demagnetization in the rotating frame
ARRF: adiabatic remagnetization in the rotating frame
BRAIN-CP: broadband adiabatic inversion cross-polarization
CEST: chemical exchange saturation transfer
CP: cross-polarization
CPMG: Carr–Purcell Meiboom–Gill
SD: spin diffusion
SNR: signal-to-noise ratio
WURST: wideband uniform-rate smooth truncation
