# Supplementary material for: Sensitivity Enhancement by Progressive Saturation of the Proton Reservoir: A Solid-State NMR Analogue of Chemical Exchange Saturation Transfer
Source: J Am Chem Soc. 2021 Nov 18;143(47):19778–84. doi: 10.1021/jacs.1c08277 (PMC8640991; doi:10.1021/jacs.1c08277)
Supplement: Supplementary file 1 — ja1c08277_si_001.pdf [file ja1c08277_si_001.pdf]

Supporting Information for

Sensitivity Enhancement by Progressive Saturation of the Proton Reservoir: A Solid-State NMR Analogue of Chemical Exchange Saturation Transfer

Michael J. Jaroszewicz,[1] Adam R. Altenhof,[2,3] Robert W. Schurko,[2,3*] and Lucio Frydman[1,3]*

[1]Department of Chemical and Biological Physics, Weizmann Institute of Science, Rehovot, Israel, 7610001.

[2]Department of Chemistry and Biochemistry, Florida State University, Tallahassee, FL 32306, USA

[3]National High Magnetic Field Laboratory, 1800 East Paul Dirac Drive, Tallahassee, FL 32310, USA

*Author to whom correspondence should be addressed. E-mail: lucio.frydman@weizmann.ac.il

Supplementary Note 1: Experimental Details and Optimization of PROSPR NMR Experiments

S1.1 Samples and Solid-State NMR Spectroscopy

Samples. Naturally-abundant samples of ammonium sulphate and *cis*-diamminedichloroplatinum (cisplatin) were purchased from Avantor (VWR) and Sigma Aldrich, respectively and used as received without further modification. The identities and purities of the samples were verified through comparisons with previously reported NMR spectra.[1,2] Both samples were ground into fine powders and *ca*. 80 mg of each was packed into a 4 mm outer-diameter glass tube (3.2 mm inner diameter) that was sealed with Teflon tape.

Solid-State NMR Spectroscopy. All NMR spectra were acquired using a Varian VNMRS console interfaced with an Oxford 14.1 T wide-bore magnet. A modified Chemagnetics HXY triple-resonance probe equipped with a 4 mm solenoid coil (1 cm long, 7 turns) devoid of ^{1}H background signals and oriented perpendicular to the $\mathbf{B}_0$ field was used to acquire all NMR spectra. All data were collected under static conditions (*i.e.*, no magic-angle spinning), with the sample temperature regulated to 20° C (unless stated otherwise). Pulse-width calibrations were carried out using either solution-state or solid-state setup samples as outlined in **Table S1**; the corresponding nutation datasets and resulting RF power calibration curves are presented in **Figure S1**. ^{33}S, ^{17}O, and ^{15}N pulses were calibrated by using ^{14}N, ^{139}La, and ^{35}Cl, respectively, and then scaling the resulting calibration curves according to the ratio between the two gyromagnetic ratios. All NMR spectra are referenced with respect to the targeted X nucleus nominal Larmor frequency, which was set to 0 kHz. Recycle delays (denoted D1) were set based on the T_1 relaxation time (D1 = $1.3\times T_1$) of the excited nucleus (either ^{1}H in ammonium sulphate and cisplatin for both PROSPR and BRAIN-CP experiments or ^{14}N in ammonium sulphate for WCPMG experiments). These were measured using a broadband analogue of saturation recovery shown in the inset of **Figure S2**, whereby a train of 20 WURST-80 pulses sweeping 500 kHz were used for saturation followed by either a single hard-pulse readout (used for ^{1}H), or a WCPMG echo-train readout (used for ^{14}N). All T_1 recovery curves (**Figure S2**) were fit with the standard mono-exponential recovery equation: $1-\exp(-\tau/T_1)$. Cross polarization experiments based on ^{1}H adiabatic demagnetization in the rotating frame (denoted ADRF-CP[3,4]) were carried out using the same pulse parameters as optimized in corresponding PROSPR NMR experiments. BRAIN-CP[5] and WCPMG[6,7]

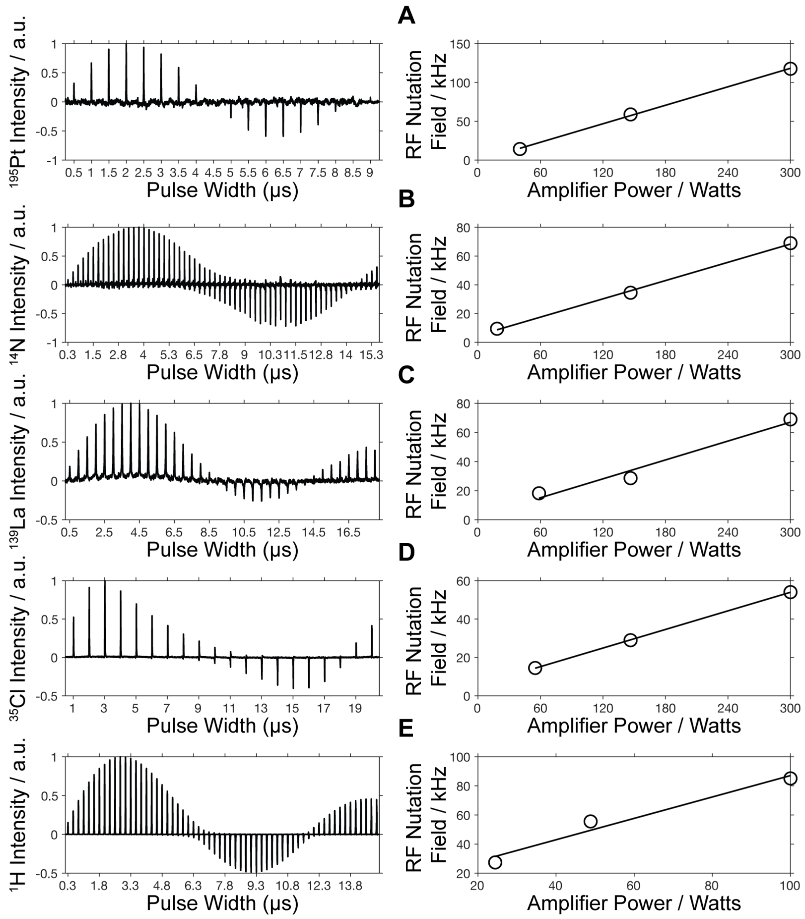

Figure S1. Left: Experimental nutation curves for (**A**) ^{195}Pt, (**B**) ^{14}N (also surrogate for ^{33}S), (**C**) ^{139}La (surrogate for ^{17}O), (**D**) ^{35}Cl (surrogate for ^{15}N), and (**E**) ^{1}H. **Right:** Corresponding RF field calibration curves showing the nutation fields measured as a function of the amplifier power (up to 300 and 100 W for the *X*- and ^{1}H-channel, respectively).

Table S1: Samples Used for Calibrating Pulse Widths

Nucleus	Sample for Pulse Width Calibration	γ-based Scaling Factor
^{1}H	Ammonium sulphate (s)<br>Cisplatin (s)	N.A.
^{195}Pt	0.5 M cisplatin (aq) in DMSO	N.A.
^{14}N	NH_4Cl (s)	N.A
^{33}S *via* ^{14}N	NH_4Cl (s)	1.0856
^{17}O *via* ^{139}La	1.0 M $LaCl_3$ (aq) in DMSO	0.9596
^{15}N *via* ^{35}Cl	NaCl (s)	1.0345

experiments were all optimized to deliver datasets with the highest signal-to-noise ratio (SNR), as well as the most uniform possible excitation across the entire breadth of the pattern. Phase cycling was not implemented in PROSPR and ADRF-CP experiments; instead, a train of WURST-80 saturation pulses was used to suppress any longitudinal *X* spin polarization immediately after the

D1 period (**Figure S3A**, **S3B**). 32 steady-state (dummy) scans were applied before the first scan of the actual variable-offset PROSPR acquisitions, in order to minimize deleterious probe instabilities resulting from RF-induced temperature fluctuations and/or the T_1 recovery of ^{1}H spin polarization. The ^{14}N PROSPR spectra shown in **Figure 2B** of the main text were acquired with the probe's *X* channel tuned and matched to the frequencies of the two "horn" discontinuities, which were coadded (additional details pertaining to the processing of ultra wideline NMR spectra are provided below). Matching to the low-frequency horn discontinuity at *ca.* −350 kHz and then to the high-frequency discontinuity *ca.* +350 kHz (also shown in **Figure 2B**, lower traces) results in lopsided appearances of the ^{14}N pattern, whereby the most intense spectral discontinuity corresponds to the frequency at which the *X* channel is tuned and matched. Nevertheless, all ^{14}N discontinuities are visible in both pieces and a uniformly excited ^{14}N PROSPR spectrum is recovered after adding these two pieces together in the frequency domain (**Figure 2B**). It is noted that a similar spectrum can be reconstructed by conventional spectral mirroring[8] involving either of the two lopsided sub-spectra. The ^{195}Pt PROSPR spectrum (**Figure 2A**) was acquired with the probe's *X* channel tuned and matched solely at the frequency corresponding to the most intense

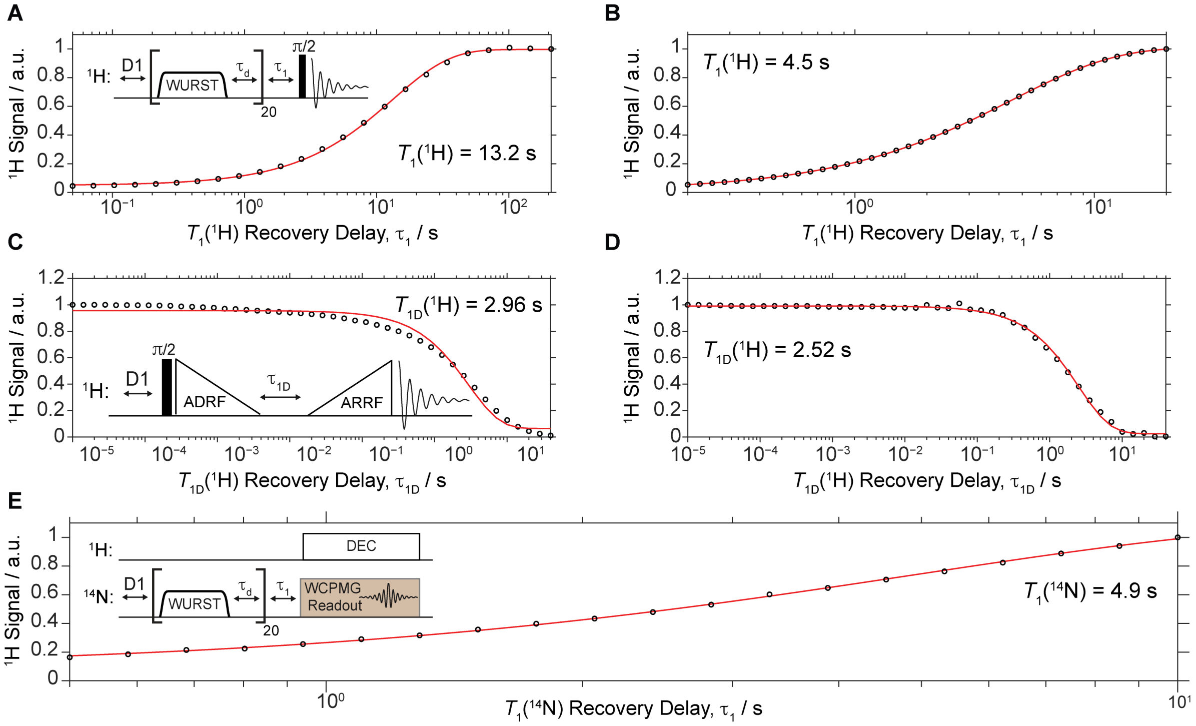

Figure S2. Experimental (**A, B**) $T_1(^{1}H)$, (**E**) $T_1(^{14}N)$, and (**C, D**) $T_{1D}(^{1}H)$ measurements acquired with the pulse sequences shown in the insets. Datasets acquired for samples of cisplatin and ammonium sulphate are shown in (**A**, **C**) and (**B**, **D**, **E**), respectively. For all T_1 and T_{1D} measurements, the saturation recovery and decay curves, respectively were fit with a mono-exponential, yielding the T_1 and T_{1D} constants provided in the respective panels.

spectral discontinuity (*ca.* −380 kHz). Likewise, all PROSPR spectra shown in **Figure 3** of the main text were acquired with the probe's *X* channel tuned and matched to the frequency resonant with the most intense part of the *X* NMR spectrum. As these data demonstrate, high-quality *X* NMR line shapes can be acquired using PROSPR without the need to re-tune the *X* channel across the entire breadth of the targeted powder pattern, as is common in frequency-stepped acquisitions involving direct detection.[9–12] Additional experimental details concerning pulse sequence

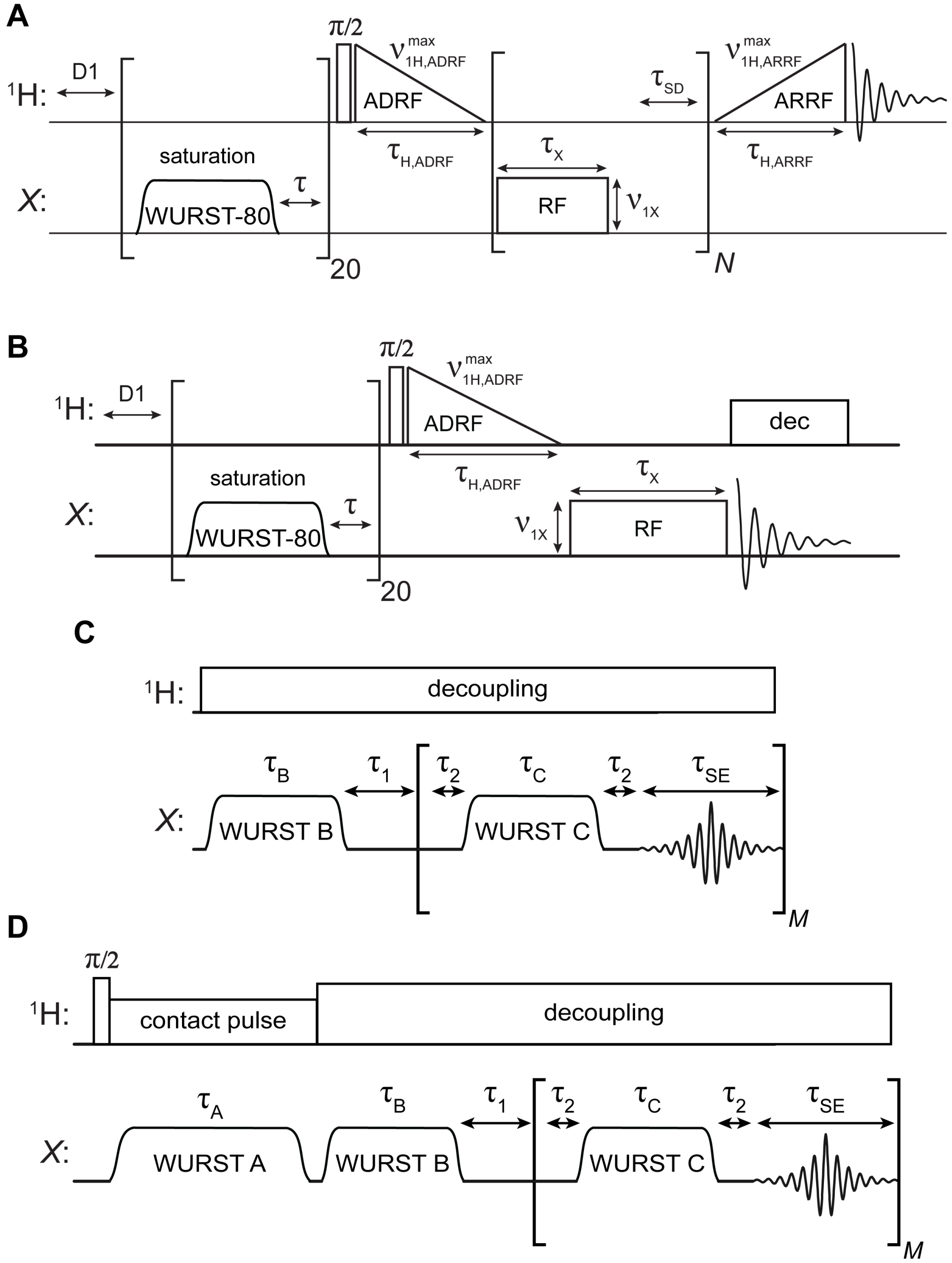

Figure S3. Schematic representations of the (**A**) PROSPR, (**B**) ADRF-CP, (**C**) WCPMG, and (**D**) BRAIN-CP pulse sequences. The shorthand variables used to denote each of the pulse/delay parameters are indicated.

parameters are contained within **Tables S2-S13** and schematic representations of all the RF pulse sequences are given in **Figure S3**.

S1.2 Data Processing and Analysis

All datasets were processed in MATLAB using custom-written processing routines. Time-domain T_2^{eff} echo trains acquired with WCPMG and BRAIN-CP were loaded into MATLAB; each echo was individually multiplied by 500 Hz of Gaussian line broadening centered at the point of maximal signal intensity, followed by multiplication with 200 Hz of Lorentzian line broadening. At this point, time-domain echo trains were either Fourier transformed with respect to t and magnitude processed, or the time-domain echo train was spliced into individual echoes that were co-added together prior to Fourier transformation and magnitude processed; these two procedures give the so-called spikelet and echo-added spectra, respectively. All WCPMG and BRAIN-CP datasets were zero-filled once. The ^{195}Pt (**Figure 2A**) and ^{14}N (**Figure 2B**) BRAIN-CP spectra were mapped out using frequency-stepped acquisition[11,12] in which each pattern was assembled by the frequency-domain co-addition of 7 and 8 individual pieces, respectively, that were acquired in *ca*. 107 kHz increments. Time-domain ^{1}H PROSPR FIDs were apodized with 100 Hz of exponential line broadening, zero-filled once to 256 points, Fourier transformed with respect to t, and absorptively phased using a zeroth-order phase correction. Plotting these ^{1}H NMR spectra as a function of the X RF transmitter offset frequency provided PROSPR's "z spectrum" (*i.e.*, the spectrum acquired by observing the intensity of a reporting resonance as a function of the frequency of a selective saturation pulse that is applied to another resonance),[13] which can be post-processed to give conventional spectral representations (**Figures 1**, **2**). This involves subtracting from each ^{1}H PROSPR NMR spectrum acquired with the X CP pulse off (denoted as S_0) the ^{1}H PROSPR NMR spectrum that was acquired with the X CP on (denoted as S). This difference divided by S_0 reveals the % of the ^{1}H NMR resonance that was attenuated/saturated and provides a semi-quantitative measure of the signal enhancement. The SNR of PROSPR NMR spectra was measured by dividing the point of maximal signal enhancement by the standard deviation of a spectral noise region devoid of genuine NMR signals. Further examples of this processing procedure are provided in the subsequent section.

S1.3 PROSPR NMR Experiments: Sequence and Optimization Procedure

Setting up and optimizing PROSPR NMR experiments is straightforward and often rapid. Step 1 involves measuring the longitudinal relaxation time-constants for the ^{1}H Zeeman order T_1(^{1}H) and the ^{1}H-^{1}H dipolar order T_{1D}(^{1}H), by using the RF pulse sequences shown in the insets of **Figures S2A** and **S2C**. Either saturation or broadband inversion recovery[14] are suitable for measuring T_1(^{1}H) (**Figures S2A**, **S2B**), whereas the value of T_{1D}(^{1}H) is obtained by first producing ^{1}H-^{1}H dipolar order *via* ADRF, which then relaxes over an incremented time τ_{1D}, and is finally remagnetized into transverse Zeeman order and readout with an adiabatic remagnetization pulse in the rotating frame (ARRF, **Figure S2C**, **S2D**). The value of T_1(^{1}H) sets an upper bound on T_{1D}(^{1}H) and is useful for setting an optimized recycle delay D1 (*i.e.*, D1 = 1.3×T_1(^{1}H)), whereas a sufficiently long T_{1D}(^{1}H) is critical for indirectly detecting the *X* NMR spectrum on the ^{1}H channel *via* PROSPR. In principle, T_{1D}(^{1}H) should be as long as possible, in order to permit depolarization (*via* CP) and repolarization (*via* spin diffusion) of the ^{1}H-^{1}H dipolar order; in practice T_{1D}(^{1}H) – and T_1(^{1}H) – should be > $N\times\tau_{SD}$, where N is the number of PROSPR loops and τ_{SD} is the ^{1}H-^{1}H spin diffusion delay (**Figure S3A**).

After setting D1 = 1.3×T_1(^{1}H) and ensuring T_{1D}(^{1}H) is sufficiently long, step 2 involves calibrating the ^{1}H ADRF and ARRF pulses. This involves optimizing the time-dependent amplitude profiles (*e.g.*, linear ramp or hyperbolic secant), overall pulse widths ($\tau_{H,ADRF}/\tau_{H,ARRF}$) and maximum RF field strengths ($\nu^{max}_{1H,ADRF}/\nu^{max}_{1H,ARRF}$), which is accomplished using the pulse sequence in **Figure S3A** with the *X* channel turned off, τ_{SD} = 0, and N = 1. In principle, a variety of *X*-channel amplitude profiles are suitable for performing heteronuclear polarization transfer; we opted for the simplest rectangular *X* pulse of constant phase and amplitude. When optimizing ^{1}H ADRF, the pulse parameters should be calibrated so as to efficiently transfer as much of the equilibrium ^{1}H Zeeman order into ^{1}H-^{1}H dipolar order; this can be carried out by minimizing the signal intensity of the ^{1}H NMR spectrum detected immediately after ADRF. The optimal ARRF conditions are identical to the optimal ADRF ones, but with the ADRF amplitude profile time reversed. We found that a simple linear ramp of the maximum available ^{1}H RF field (in our case *ca*. 70 kHz with ~100 W) applied with constant phase over the course of $\tau_{H,ADRF}$ = 10 ms was sufficient for transferring >90% of the Zeeman order into dipolar order in all of the cases investigated herein. It is noted that the ^{1}H ADRF and ARRF ramp pulses are extremely sensitive

to resonance offsets, and it is therefore important that the ^{1}H carrier frequency be precisely set and resonant with the frequency corresponding to maximal ^{1}H signal intensity.

Step 3 involves optimizing the ^{1}H-*X* dipolar order-driven multiple-contact CP conditions. This is accomplished by adjusting the *X*-channel RF field strength (ν_{1X}) and pulse width (τ_X), whilst monitoring the corresponding attenuation of the ^{1}H signal intensity for an arbitrary *N* and τ_{SD}. We found that initial unoptimized values of $N = 40\text{-}50$ and $\tau_{SD} = 1\text{-}20$ ms were sufficient for discerning a detectable reduction of ^{1}H signal intensity, which can be visualized by plotting the difference between the maximum ^{1}H signal intensities measured with the *X*-channel off (S_0) and with the *X*-channel on (*S*), as function of ν_{1X} and τ_X . **Figures S4A-S9A** and **Figures S4B-S9B** show plots of $(S_0{-}S)/(S_0)$ as a function of the *X* pulse parameters ν_{1X} and τ_X, respectively; the optimal parameters are those that deplete the largest % of ^{1}Hs. Prior to performing these optimization steps, it is beneficial to collect a preliminary PROSPR NMR *z* spectrum using a coarse *X* RF transmitter offset step size in order to determine the approximate *X*-channel offset frequency that gives the largest overall ^{1}H signal attenuation (*e.g.*, by using ~50 distinct *X* offset frequencies discretized over a broad region), before turning to the next step.

Step 4 involves optimizing the τ_{SD} and *N* combination that gives the largest overall attenuation of the ^{1}H resonance, which again can be visualized by plotting the % of depleted ^{1}Hs as a function of both τ_{SD} and *N,* and choosing the combination resulting in the largest % of ^{1}Hs saturated (**Figures S4C-S9C** and **Figures S4D-S9D**). For all PROSPR optimizations, 10 exponentially-spaced τ_{SD} values between 10 μs and 250 ms were used with 15 PROSPR loop variables spaced between $N = 1$ and $N = 80$. While only a single S_0 dataset is required for these optimizations, it is recommended to collect a few S_0 ^{1}H NMR spectra interleaved with the *S* acquisitions, so as to gauge the stability of the probe. In principle, all S_0 ^{1}H NMR spectra collected throughout the optimization process should be identical, with any deviation from spectrum-to-spectrum resulting in undesirable noise artifacts that will reduce potential signal enhancements; sampling several S_0 data points partially alleviates these issues. The data presented in **Figures S4C-S9C** show the S_0 (black) and *S* (red) ^{1}H NMR spectra collected over the course of the τ_{SD}/N optimization, in which the *X* channel is off and on, respectively, thereby revealing the PROSPR-based depletion of ^{1}H-^{1}H dipolar order. Calculating $(S_0{-}S)/(S_0)$ and rearranging the data so that the number of loops and spin diffusion array variables appear on orthogonal axes gives the datasets presented in **Figures S4D-S9D**.

Finally, with the optimized PROSPR pulse parameters, step 5 consists of varying the *X* RF offset frequency over the targeted *X* spectral range using a sufficiently large number of offsets. In principle, the value of ν_{1X} sets a lower bound on the *X* RF transmitter offset step size $\Delta\nu_X$, as PROSPR cannot resolve *X* spectral contributions to the ^{1}H saturation when $\Delta\nu_X << \nu_{1X}$ (**Supplement 4**). Hence, it may be desirable in some cases to use lower-than-optimal ν_{1X} powers at the expense of increasing *N*, so as to better resolve and rasterize *X* spectral discontinuities on the ^{1}H NMR spectra without sacrificing signal enhancements. In practice this was not a significant issue for the pattern breadths investigated here. Furthermore, it was noticed that retuning of the *X*-channel was not nearly as critical when stepping the *X* RF transmitter frequency in PROSPR as it was in direct *X*-nuclei acquisitions. This is a result of the broadness of ADRF-based CP matching conditions (given by a spectrum of dipolar frequencies), coupled to a further resilience against mismatches and inefficiencies of ^{1}H spin polarization transfers, provided by PROSPR's multiple, looped CP repetitions.

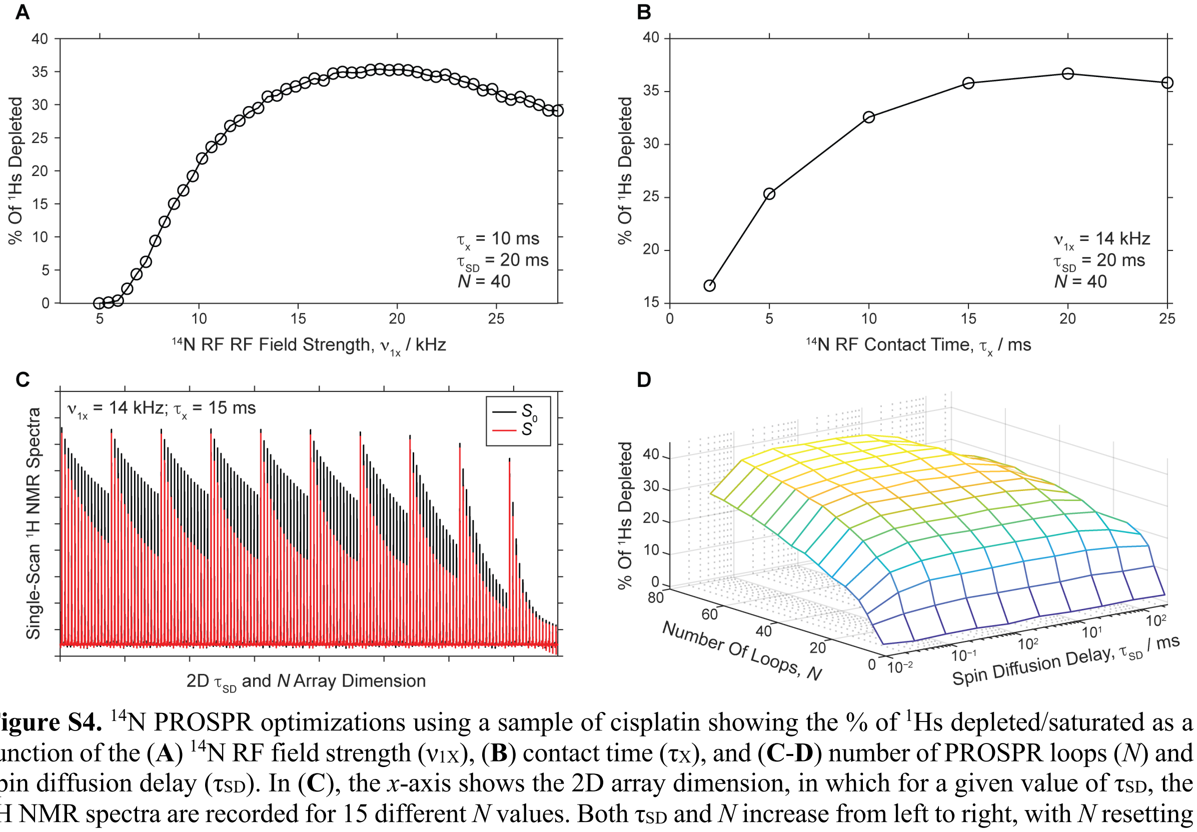

Figure S4. ^{14}N PROSPR optimizations using a sample of cisplatin showing the % of ^{1}Hs depleted/saturated as a function of the (**A**) ^{14}N RF field strength (ν_{1X}), (**B**) contact time (τ_X), and (**C**-**D**) number of PROSPR loops (*N*) and spin diffusion delay (τ_{SD}). In (**C**), the *x*-axis shows the 2D array dimension, in which for a given value of τ_{SD}, the ^{1}H NMR spectra are recorded for 15 different *N* values. Both τ_{SD} and *N* increase from left to right, with *N* resetting (*i.e.* 15 values of *N*, with *N*=1 to *N*=80) with each subsequent τ_{SD} increment. **(D)** shows the same data, after rearranging it into 2 dimensions and using it to calculate % depletion = $(S_0-S)/S_0\times100$.

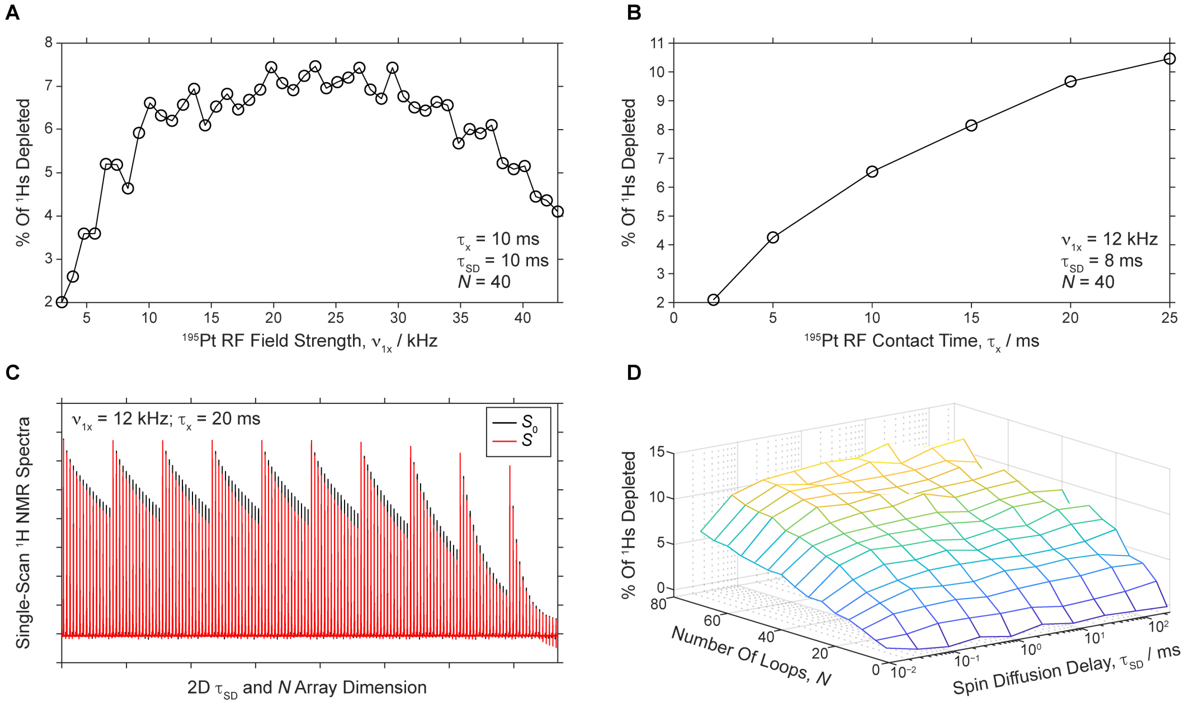

Figure S5. ^{195}Pt PROSPR optimizations using a sample of cisplatin showing the % of ^{1}Hs depleted/saturated as a function of the (**A**) ^{195}Pt RF field strength (ν_{1X}), (**B**) contact time (τ_X), and (**C**-**D**) number of PROSPR loops (N) and spin diffusion delay (τ_{SD}). The negative ^{1}H intensity observed at long τ_{SD} values results from the excitation of partially recovered ^{1}H polarization over the course of the ^{1}H ARRF pulse.

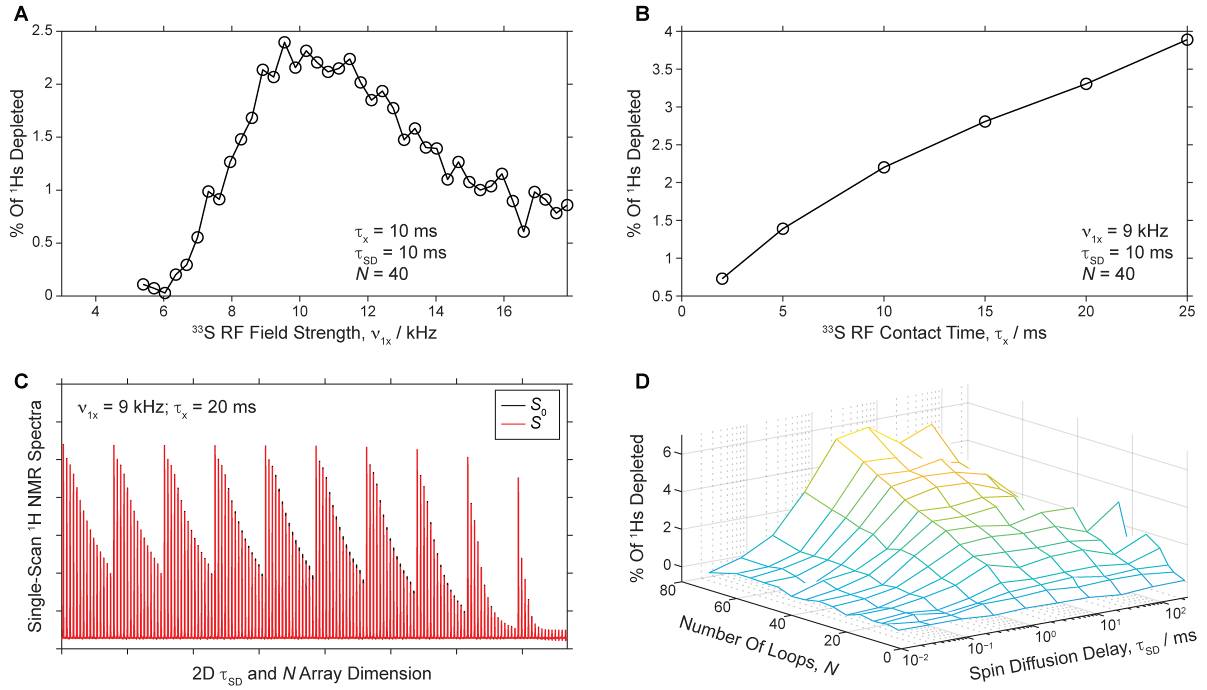

Figure S6. ^{33}S PROSPR optimizations using a sample of ammonium sulphate showing the % of ^{1}Hs depleted/saturated as a function of the (**A**) ^{33}S RF field strength (ν_{1X}), (**B**) contact time (τ_X), and (**C**-**D**) number of PROSPR loops (N) and spin diffusion delay (τ_{SD}).

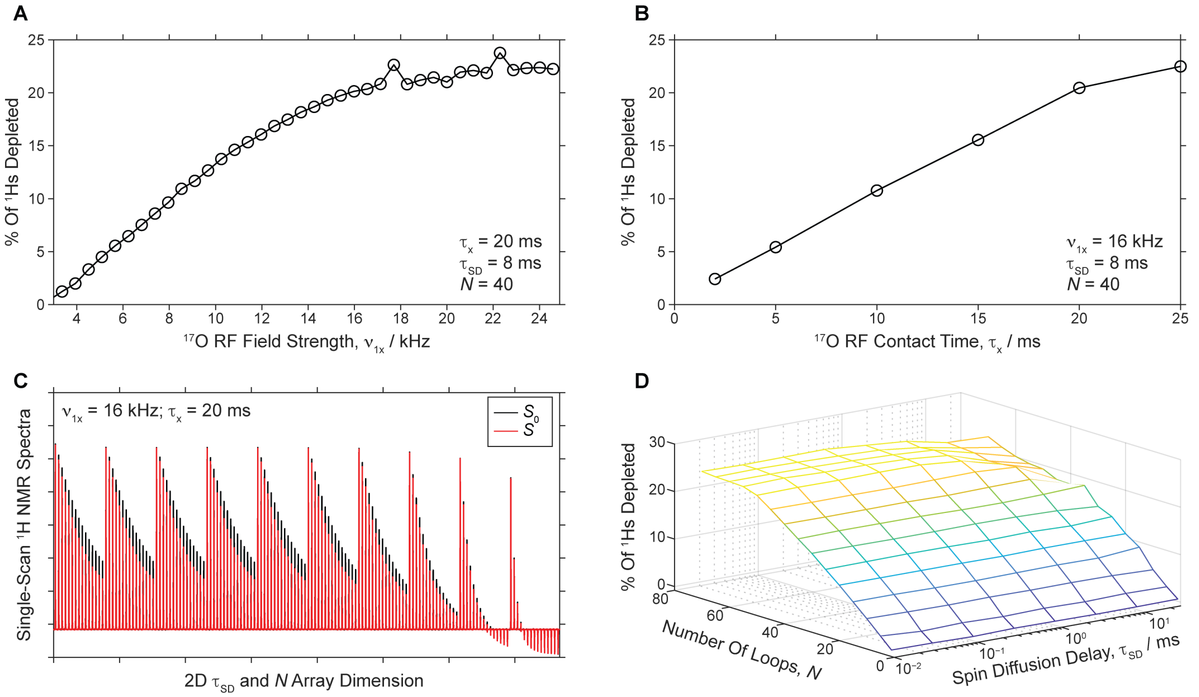

Figure S7. ^{17}O PROSPR optimizations using a sample of ammonium sulphate showing the % of ^{1}Hs depleted/saturated as a function of the (**A**) ^{17}O RF field strength (ν_{1X}), (**B**) contact time (τ_X), and (**C**-**D**) number of PROSPR loops (N) and spin diffusion delay (τ_{SD}).

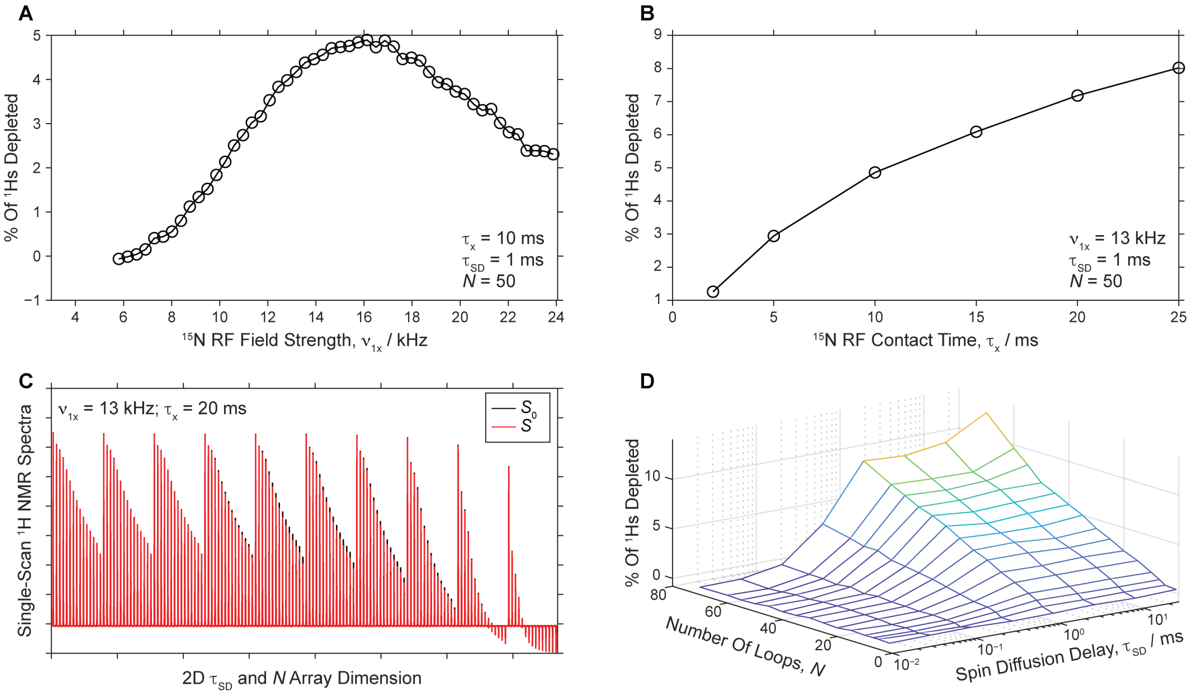

Figure S8. ^{15}N PROSPR optimizations using a sample of ammonium sulphate showing the % of ^{1}Hs depleted/saturated as a function of the (**A**) ^{15}N RF field strength (ν_{1X}), (**B**) contact time (τ_X), and (**C**-**D**) number of PROSPR loops (N) and spin diffusion delay (τ_{SD}).

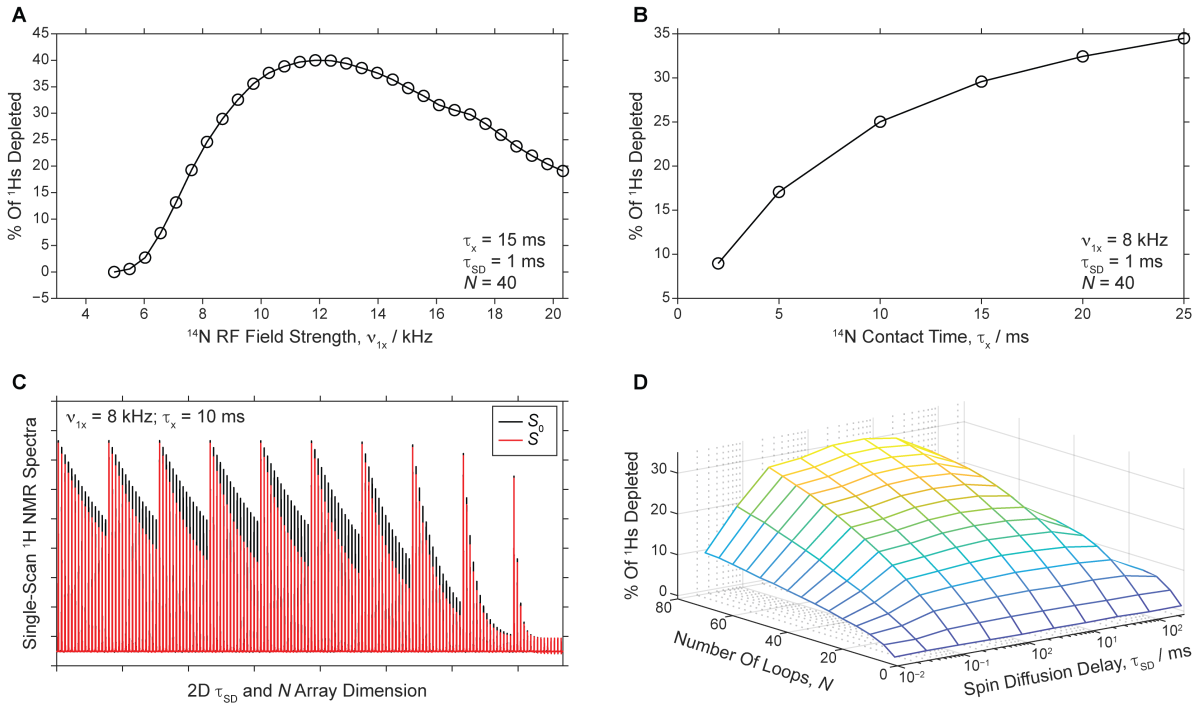

Figure S9. ^{14}N PROSPR optimizations using a sample of ammonium sulphate showing the % of ^{1}Hs depleted/saturated as a function of the (**A**) ^{14}N RF field strength (ν_{1X}), (**B**) contact time (τ_X), and (**C**-**D**) number of PROSPR loops (N) and spin diffusion delay (τ_{SD}).

Table S2: Experimental parameters for ^{195}Pt PROSPR experiments in Figure 2.

Compound	*cis*-diamminedichloroplatinum (cisplatin)
**B**$_0$ (T)	14.1
Number of Transients	4
Number of ^{195}Pt Transmitter Offsets, np1[a]	200
Recycle Delay, D1 (s)	17
Spectral Window Width (kHz)	500
Dwell Time (µs)	2
Number of PROSPR loops (N)	60
Spin Diffusion Delay, τ_{SD} (ms)	0.9
^{195}Pt PROSPR Step Size, $\Delta\nu_X$ (kHz)	8.75
^{195}Pt PROSPR Start/End Offset Frequency (kHz)	−750 / 991.25
^{1}H FID Acquisition Time (ms)	1
^{1}H Excitation Pulse Length (µs)	3.5
^{1}H Excitation Pulse Amplitude (kHz)	71
^{1}H ADRF/ARRF Pulse Length, $\tau_{H,ADRF/ARRF}$ (ms)	10
^{1}H ADRF/ARRF Max Pulse Amplitude, $\nu^{max}{}_{1H,ADRF/ARRF}$ (kHz)	71
^{195}Pt Contact Time, τ_X (ms)	20
^{195}Pt Pulse Amplitude, ν_{1X} (kHz)	12

[a] The ^{195}Pt channel was tuned and matched to a single frequency only, which corresponded to the frequency of the most intense part of the ^{195}Pt NMR spectrum (*ca.* −380 kHz). The ^{195}Pt simulation

of **Figure 2A** was generated with the following CS tensor parameters according to the Herzfeld Berger convention: Ω = 8808 ppm, κ = −0.96, and δ_{iso} = −300 ppm.

Table S3: Experimental parameters for ^{195}Pt BRAIN-CP experiments in Figure 2.

Compound	*cis*-diamminedichloroplatinum (cisplatin)
**B**$_0$ (T)	14.1
Number of Transients	72
Number of ^{195}Pt Transmitter Offsets[a]	7
Recycle Delay, D1 (s)	17
Spectral Window Width (kHz)	2500
Dwell Time (μs)	0.4
Number of Meiboom-Gill loops (*M*)	80
Spin Echo Length (μs)	102.4
Acquisition Time (ms)	8.192
^{1}H Excitation Pulse Length (μs)	3.5
^{1}H Excitation Pulse Amplitude (kHz)	71
^{1}H CP Pulse Amplitude (kHz)	40
CP Contact Time, τ_A (ms)	10
WURST-A Amplitude $\nu_{1,A}$ (kHz)	34
WURST-A Sweep width, $\Delta\nu_A$ (kHz)	500
WURST-B length, τ_B (μs)	50
WURST-B Sweep width, $\Delta\nu_B$ (kHz)	500
WURST-B Amplitude $\nu_{1,B}$ (kHz)	40
WURST-C length, τ_C (μs)	50
WURST-C Sweep width, $\Delta\nu_C$ (kHz)	500
WURST-C Amplitude, $\nu_{1,C}$ (kHz)	40
^{1}H CW Decoupling RF Power (kHz)	42

[a] The ^{195}Pt channel was re-tuned at every ^{195}Pt transmitter offset frequency.

Table S4: Experimental parameters for ^{14}N PROSPR experiments in Figure 2.

Compound	*cis*-diamminedichloroplatinum (cisplatin)
**B**$_0$ (T)	14.1
Number of Transients	1
Number of ^{14}N Transmitter Offsets, np1[a]	200
Recycle Delay, D1 (s)	17
Spectral Window Width (kHz)	500
Dwell Time (μs)	2
Number of PROSPR loops (*N*)	65
Spin Diffusion Delay, τ_{SD} (ms)	0.29
^{14}N PROSPR Step Size, $\Delta\nu_X$ (kHz)	10
^{14}N PROSPR Start/End Offset Frequency (kHz)	−1000 / 990
^{1}H FID Acquisition Time (ms)	1
^{1}H Excitation Pulse Length (μs)	3.5
^{1}H Excitation Pulse Amplitude (kHz)	71

^{1}H ADRF/ARRF Pulse Length, $\tau_{H,ADRF/ARRF}$ (ms)	10
^{1}H ADRF/ARRF Max Pulse Amplitude, $\nu^{max}{}_{1H,ADRF/ARRF}$ (kHz)	71
^{14}N Contact Time, τ_X (ms)	15
^{14}N Pulse Amplitude, ν_{1X} (kHz)	14

[a] The ^{14}N channel was tuned and matched at two distinct frequencies corresponding to the two horn discontinuities (at *ca*. ± 350 kHz) of the ^{14}N pattern, which gave two ^{1}H-^{14}N PROSPR NMR spectra. The ^{14}N simulation of **Figure 2B** was generated with the following EFG tensor parameters: C_Q = 0.99 MHz, η_Q = 0.12, and δ_{iso} = 0 ppm.

Table S5: Experimental parameters for ^{14}N BRAIN-CP experiments in Figure 2.

Compound	*cis*-diamminedichloroplatinum (cisplatin)
$\mathbf{B}_0$ (T)	14.1
Number of Transients	208
Number of ^{14}N Transmitter Offsets[a]	8
Recycle Delay, D1 (s)	17
Spectral Window Width (kHz)	2500
Dwell Time (μs)	0.4
Number of Meiboom-Gill loops (*M*)	200
Spin Echo Length (μs)	102.4
Acquisition Time (ms)	20.48
^{1}H Excitation Pulse Length (μs)	3.5
^{1}H Excitation Pulse Amplitude (kHz)	71
^{1}H CP Pulse Amplitude (kHz)	40
CP Contact Time, τ_A (ms)	10
WURST-A Amplitude $\nu_{1,A}$ (kHz)	26
WURST-A Sweep width, $\Delta\nu_A$ (kHz)	500
WURST-B length, τ_B (μs)	50
WURST-B Sweep width, $\Delta\nu_B$ (kHz)	500
WURST-B Amplitude $\nu_{1,B}$ (kHz)	35
WURST-C length, τ_C (μs)	50
WURST-C Sweep width, $\Delta\nu_C$ (kHz)	500
WURST-C Amplitude, $\nu_{1,C}$ (kHz)	35
^{1}H CW Decoupling RF Power (kHz)	42

[a] The ^{14}N channel was re-tuned at every ^{14}N transmitter offset frequency.

Table S6: Experimental parameters for ^{33}S PROSPR experiments in Figure 3.

Compound	Ammonium Sulphate
$\mathbf{B}_0$ (T)	14.1
Number of Transients	1
Number of ^{33}S Transmitter Offsets, np1	50
Recycle Delay, D1 (s)	6
Spectral Window Width (kHz)	500
Dwell Time (μs)	2
Number of PROSPR loops (*N*)	70
Spin Diffusion Delay, τ_{SD} (ms)	2.7
^{33}S PROSPR Step Size, $\Delta\nu_X$ (kHz)	2

^{33}S PROSPR Start/End Offset Frequency (kHz)	92 / −8
^{1}H FID Acquisition Time (ms)	1
^{1}H Excitation Pulse Length (μs)	3.5
^{1}H Excitation Pulse Amplitude (kHz)	71
^{1}H ADRF/ARRF Pulse Length, $\tau_{H,ADRF/ARRF}$ (ms)	10
^{1}H ADRF/ARRF Max Pulse Amplitude, $\nu^{max}_{1H,ADRF/ARRF}$ (kHz)	71
^{33}S Contact Time, τ_X (ms)	20
^{33}S Pulse Amplitude, ν_{1X} (kHz)	9

Table S7: Experimental parameters for ^{33}S ADRF-CP experiments in Figure 3.

Compound	Ammonium Sulphate
**B**$_0$ (T)	14.1
Number of Transients	50
Recycle Delay, D1 (s)	6
Spectral Window Width (kHz)	100
Dwell Time (μs)	10
^{33}S FID Acquisition Time (ms)	5
^{1}H Excitation Pulse Length (μs)	3.5
^{1}H Excitation Pulse Amplitude (kHz)	71
^{1}H ADRF Pulse Length, $\tau_{H,ADRF}$ (ms)	10
^{33}S Contact Time, τ_X (ms)	20
^{33}S Pulse Amplitude, ν_{1X} (kHz)	9
^{1}H CW Decoupling RF Power (kHz)	35

Table S8: Experimental parameters for ^{17}O PROSPR experiments in Figure 3.

Compound	Ammonium Sulphate
**B**$_0$ (T)	14.1
Number of Transients	1
Number of ^{17}O Transmitter Offsets, np1	100
Recycle Delay, D1 (s)	6
Spectral Window Width (kHz)	500
Dwell Time (μs)	2
Number of PROSPR loops (*N*)	70
Spin Diffusion Delay, τ_{SD} (ms)	0.1
^{17}O PROSPR Step Size, $\Delta\nu_X$ (kHz)	20
^{17}O PROSPR Start/End Offset Frequency (kHz)	−1000/ 980
^{1}H FID Acquisition Time (ms)	1
^{1}H Excitation Pulse Length (μs)	3.5
^{1}H Excitation Pulse Amplitude (kHz)	71
^{1}H ADRF/ARRF Pulse Length, $\tau_{H,ADRF/ARRF}$ (ms)	10
^{1}H ADRF/ARRF Max Pulse Amplitude, $\nu^{max}_{1H,ADRF/ARRF}$ (kHz)	71
^{17}O Contact Time, τ_X (ms)	20
^{17}O Pulse Amplitude, ν_{1X} (kHz)	16

Table S9: Experimental parameters for ^{17}O ADRF-CP experiments in Figure 3.

Compound	Ammonium Sulphate
$\mathbf{B_0}$ (T)	14.1
Number of Transients	50
Recycle Delay, D1 (s)	6
Spectral Window Width (kHz)	2500
Dwell Time (μs)	0.4
^{17}O FID Acquisition Time (ms)	5
^{1}H Excitation Pulse Length (μs)	3.5
^{1}H Excitation Pulse Amplitude (kHz)	71
^{1}H ADRF Pulse Length, $\tau_{H,ADRF}$ (ms)	10
^{17}O Contact Time, τ_X (ms)	20
^{17}O Pulse Amplitude, ν_{1X} (kHz)	16
^{1}H CW Decoupling RF Power (kHz)	35

Table S10: Experimental parameters for ^{15}N PROSPR experiments in Figure 3.

Compound	Ammonium Sulphate
$\mathbf{B_0}$ (T)	14.1
Number of Transients	1
Number of ^{15}N Transmitter Offsets, np1	50
Recycle Delay, D1 (s)	6
Spectral Window Width (kHz)	500
Dwell Time (μs)	2
Number of PROSPR loops (N)	70
Spin Diffusion Delay, τ_{SD} (ms)	8
^{15}N PROSPR Step Size, $\Delta\nu_X$ (kHz)	2
^{15}N PROSPR Start/End Offset Frequency (kHz)	−50/ 48
^{1}H FID Acquisition Time (ms)	1
^{1}H Excitation Pulse Length (μs)	3.5
^{1}H Excitation Pulse Amplitude (kHz)	71
^{1}H ADRF/ARRF Pulse Length, $\tau_{H,ADRF/ARRF}$ (ms)	10
^{1}H ADRF/ARRF Max Pulse Amplitude, $\nu^{max}{}_{1H,ADRF/ARRF}$ (kHz)	71
^{15}N Contact Time, τ_X (ms)	20
^{15}N Pulse Amplitude, ν_{1X} (kHz)	13

Table S11: Experimental parameters for ^{15}N ADRF-CP experiments in Figure 3.

Compound	Ammonium Sulphate
$\mathbf{B_0}$ (T)	14.1
Number of Transients	50
Recycle Delay (s), D1	6
Spectral Window Width (kHz)	100
Dwell Time (μs)	10
^{15}N FID Acquisition Time (ms)	5
^{1}H Excitation Pulse Length (μs)	3.5
^{1}H Excitation Pulse Amplitude (kHz)	71
^{1}H ADRF Pulse Length, $\tau_{H,ADRF}$ (ms)	10

^{15}N Contact Time, τ_X (ms)	20
^{15}N Pulse Amplitude, ν_{1X} (kHz)	13
^{1}H CW Decoupling RF Power (kHz)	35

Table S12: Experimental parameters for ^{14}N PROSPR experiments in Figure 3.

Compound	Ammonium Sulphate
$\mathbf{B}_0$ (T)	14.1
Number of Transients	1
Number of ^{14}N Transmitter Offsets, np1	250
Recycle Delay, D1 (s)	6
Spectral Window Width (kHz)	500
Dwell Time (μs)	2
Number of PROSPR loops (N)	70
Spin Diffusion Delay, τ_{SD} (ms)	1
^{14}N PROSPR Step Size, $\Delta\nu_X$ (kHz)	2
^{14}N PROSPR Start/End Offset Frequency (kHz)	−250/ 248
^{1}H FID Acquisition Time (ms)	1
^{1}H Excitation Pulse Length (μs)	3.5
^{1}H Excitation Pulse Amplitude (kHz)	71
^{1}H ADRF/ARRF Pulse Length, $\tau_{H,ADRF/ARRF}$ (ms)	10
^{1}H ADRF/ARRF Max Pulse Amplitude, $\nu^{max}{}_{1H,ADRF/ARRF}$ (kHz)	71
^{14}N Contact Time, τ_X (ms)	10
^{14}N Pulse Amplitude, ν_{1X} (kHz)	8

The ^{14}N simulation of **Figure 3** was generated with the following EFG tensor parameters: $C_Q(1)$ = 150 kHz, $\eta_Q(1)$ = 0.7, and $\delta_{iso}(1)$ = 0 ppm; $C_Q(2)$ = 110 kHz, $\eta_Q(2)$ = 0.75, and $\delta_{iso}(2)$ = 0 ppm.

Table S13: Experimental parameters for ^{14}N WCPMG experiments in Figure 3.

Compound	Ammonium Sulphate
$\mathbf{B}_0$ (T)	14.1
Number of Transients	1208
Recycle Delay, D1 (s)	6
Spectral Window Width (kHz)	2500
Dwell Time (μs)	0.4
Number of Meiboom-Gill loops (M)	80
Spin Echo Length, τ_{SE} (μs)	409.6
Acquisition Time (ms)	32.76
WURST-B length, τ_B (μs)	50
WURST-B Sweep width, $\Delta\nu_B$ (kHz)	1000
WURST-B Amplitude $\nu_{1,B}$ (kHz)	21
WURST-C length, τ_C (μs)	50
WURST-C Sweep width, $\Delta\nu_C$ (kHz)	1000
WURST-C Amplitude, $\nu_{1,C}$ (kHz)	21
^{1}H CW Decoupling RF Power (kHz)	35

Supplementary Note 2: RF-Driven Decay of the Proton Resonance Upon Looping a Conventional CP

The PROSPR experiments presented here exploit ^{1}H-^{1}H dipolar order, rather than ^{1}H Zeeman order, for sensitivity enhancement. PROSPR experiments could also be designed based on looped CP modules involving the repeated transfer of abundant ^{1}H Zeeman order.[15–18] In fact, our preliminary tests were based on a conventional looped CP pulse sequence, consisting of an initial excitation of equilibrium ^{1}H Zeeman order, followed by its coherent transfer to heteronuclear dipolar-coupled spins *via* the simultaneous application of CP spin-locking pulses. After this, a phase-shifted $\pi/2$ flip-back pulse stores the remaining transverse ^{1}H spin polarization (largely originating from X-decoupled ^{1}Hs) along the longitudinal $+z$ axis of the rotating frame, and a subsequent free evolution period (denoted with τ_{SD}) allows the saturated ^{1}H spin polarization to undergo spin diffusion-driven repolarization from fully z-polarized X-decoupled ^{1}H spins,[19] while the polarized X spins rapidly dephase. These steps were then repeated N times prior to ^{1}H detection. Unlike the dipolar order-based method discussed in the main text, however, we found that each ^{1}H spin-locking repetition is associated with substantial ^{1}H signal intensity losses.[20,21]

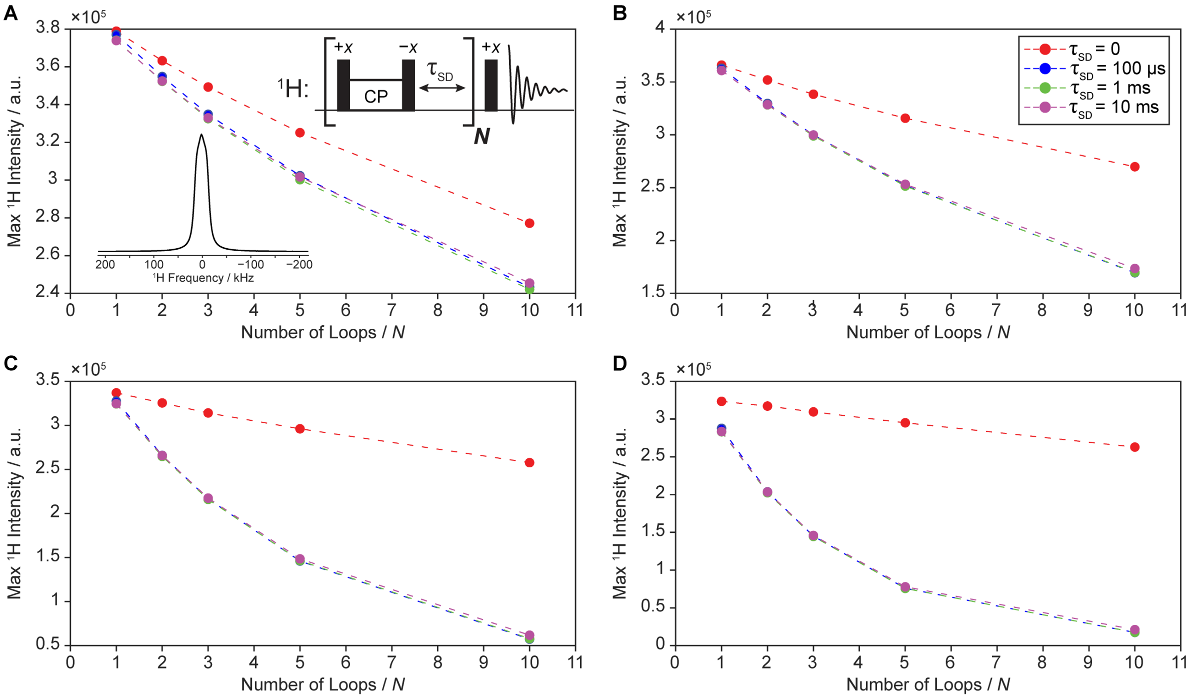

Figure S10. The ^{1}H signal intensity of ammonium sulphate plotted as a function of the number of loops (N) for different spin diffusion delays (τ_{SD}, colours shown in the legend) and ^{1}H spin-locking RF fields of $\nu_{1,SL}(^{1}\text{H})$ = (**A**) 75 kHz, (**B**) 56 kHz, (**C**) 39 kHz, and (**D**) 22 kHz. The RF pulse sequence used to collect these data is shown in the inset. The solid rectangles refer to calibrated $\pi/2$ pulses.

These deleterious effects complicate the realization of ^{1}H Zeeman order-based PROSPR, unless experiments are executed with high RF fields. **Figure S10** shows this for the ^{1}H NMR resonance of ammonium sulphate as an experimental example. The evolution of the proton signal intensity is here plotted as a function of the number of loops (N) at different τ_{SD} times (shown in different colours) and at four distinct ^{1}H spin-locking RF field strengths (corresponding to each panel). **Figure S10A** shows that after $N = 10$ loops (*i.e.*, 10 spin-locking elements), the ^{1}H signal decreases by *ca*. 25% from its value at $N = 1$ when $\tau_{SD} = 0$ (red curve) and when using a ^{1}H spin-locking RF field strength of $\nu_{1,SL}(^{1}H) = 75$ kHz. For non-zero evolution times however, *ca*. 37% of the ^{1}H signal decays when $N = 10$ for all τ_{SD} values tested. These signal losses become progressively larger as the strength of the ^{1}H spin-locking pulse decreases, as demonstrated by the plots in the other three panels. While these losses may be reduced by employing very high ^{1}H RF fields for spinning locking, this necessitates commensurately high RF powers on the X-channel in order to satisfy Hartmann-Hahn matching, which in most cases is not feasible – especially when targeting low-γ X nuclei (*e.g.*, ^{14}N, ^{33}S). This would also lead to unacceptably high probe duty cycles and poor ^{1}H-detected X spectral resolution in subsequent PROSPR spectra (see **Supplement 4** for a more in-depth discussion regarding this last point). It is worth mentioning that these deleterious effects are largely mitigated under magic-angle spinning, as will be discussed in a forthcoming publication.

Supplementary Note 3: Numerical Simulations of PROSPR's Dipolar-Order Spin Dynamics

This section presents and discusses numerical simulations that examine and corroborate the multi-step, saturation-based PROSPR mechanism outlined in **Figure 1** of the main text. These custom-written MATLAB-based simulations were performed in Hilbert space using nine spin-1/2 nuclei (one ^{195}Pt and eight ^{1}Hs) that were distributed on a planar grid and organized into three distinct pools on the basis of heteronuclear dipolar coupling (inset, **Figure S11A**). A single ^{195}Pt spin occupies the dilute X pool (shown in red), which is heteronuclear-dipolar coupled to one ^{1}H only (occupying the X-coupled ^{1}H pool shown in green) that is $r_{XH} = 1.25$ Å away, resulting in a heteronuclear dipolar coupling constant of $b = 12.8$ kHz. This single ^{1}H is in turn homonuclear-dipolar coupled to seven mutually coupled ^{1}Hs occupying an abundant pool (shown in blue) that

are separated from their nearest neighbour by r_{HH} = 1.5 Å, from which the resulting 28 ^{1}H-^{1}H dipolar couplings are calculated (the resulting dipolar-broadened ^{1}H NMR spectrum of all 8 ^{1}Hs is shown in the inset of **Figure S11C**). Notice that there is no heteronuclear dipolar coupling between the ^{195}Pt spin and the seven abundant ^{1}Hs. Thus, any transfer of spin polarization/saturation between these dipolar-decoupled pools must be mediated by the single ^{1}H that occupies the *X*-coupled pool, which is mutually coupled to both pools. This heteronuclear dipolar coupling topology therefore approximates the conditions often present in solids, where the heteronucleus, *X*, is dilute, and strongly dipolar coupled only to a small fraction of the abundant ^{1}H nuclei. These simulations do not consider the effects of relaxation.

Figure S11A shows the time-dependent trajectories of five distinct operators (indicated by the different colours) over the course of a 10 ms ^{1}H ADRF pulse, whose amplitude is modulated according to a hyperbolic secant having a maximum RF field strength of $\nu^{max}_{1H,ADRF}$ = 80 kHz (inset, **Figure S11A**). The initial density matrix at t = 0 corresponds to fully polarized equilibrium

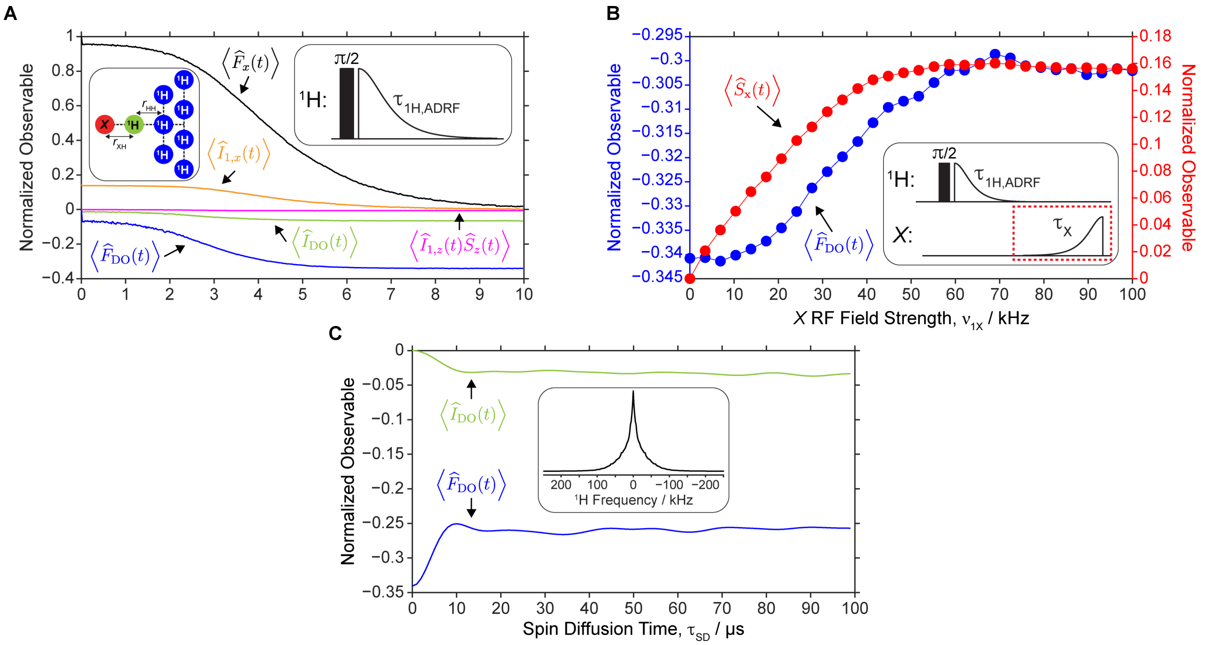

Figure S11. Hilbert space numerical simulations monitoring dipolar- and Zeeman-order (indicated in each panel) for a nine spin-1/2 system (inset, **A**) subjected to (**A**) ^{1}H ADRF and then (**B**) *X*-channel CP. (**C**) Shows the spin diffusion of dipolar order between the dipolar-coupled abundant (blue curve) and the *X*-coupled ^{1}H (green curve) spin pools over a 100 μs free evolution interval. The pulse widths for all ADRF and ARRF pulses are 10 ms, whose amplitudes are modulated according to a hyperbolic secant and its time-reversed counterpart, respectively, which have RF field strengths of $\nu^{max}_{1H,ADRF}$ = ν^{max}_{1H} = 80 kHz. All pulses are applied on resonance and the interaction Hamiltonian contains contributions from both the hetero- and homonuclear dipolar coupling interactions, as well as the applied RF. The ^{1}H NMR spectrum shown in the inset of (**C**) was simulated with 1500 crystallites sampled according to the ZCW scheme,[27,28] whilst all other simulations were carried out at a single crystallite orientation with β = 90° in which all of the internuclear dipolar vectors are coincident. See text for additional definitions.

^{1}H Zeeman order (*i.e.*, $\hat{\rho}(0) = \hat{I}_{1,z} + \sum_{i\geq 2}^{8} \hat{I}_{i,z}$, where $\hat{I}_{1,z}$ and $\hat{I}_{i,z}$ are the z-component spin-angular momentum operators of *X*-coupled (*i.e.*, i = 1) and *X*-decoupled ^{1}Hs (*i.e.*, $i \geq 2$, where the inset in **Figure S12** shows the explicit numbering of the ^{1}Hs), respectively and $\hat{S}_m$ (*vide infra*) denotes the mth component spin-angular momentum operator of the dilute ^{195}Pt spin), which is excited to the $+x$ axis of the rotating frame using the pulse sequence shown in the inset. The black and orange curves show the dynamics of the transverse spin polarization corresponding to the abundant ^{1}H reservoir (*i.e.*, $\langle \hat{F}_x(t) \rangle = \sum_{i\geq 2}^{8} \langle \hat{I}_{i,x}(t) \rangle$ where $\hat{F}_i$ is the ith component of the total spin operator for the abundant pool), and of the *X*-coupled ^{1}H (*i.e.*, $\langle \hat{I}_{1x}(t) \rangle$, respectively) over the course of ADRF, which after 10 ms are nearly completely demagnetized having expectations values of $\langle \hat{F}_x(\tau_{\text{ADRF}}) \rangle = \langle \hat{I}_{1x}(\tau_{\text{ADRF}}) \rangle = \sim 0$. The transverse Zeeman order (denoted ZO) for each ^{1}H pool is transferred into ^{1}H-^{1}H dipolar order (denoted DO) as shown by the green and blue curves, which represent the total DO existing on the *X*-coupled (*i.e.*, $\langle \hat{I}_{\text{DO}}(t) \rangle = \sum_{j=2}^{8} \langle 2\hat{I}_{1,z}(t)\hat{I}_{j,z}(t) - \hat{I}_{1,x}(t)\hat{I}_{j,x}(t) - \hat{I}_{1,y}(t)\hat{I}_{j,y}(t) \rangle$) and *X*-decoupled abundant ^{1}H pools (*i.e.*, $\langle \hat{F}_{\text{DO}}(t) \rangle = \sum_{i<j,i\neq j\neq 1}^{8} \langle 2\hat{I}_{i,z}(t)\hat{I}_{j,z}(t) - \hat{I}_{i,x}(t)\hat{I}_{j,x}(t) - \hat{I}_{i,y}(t)\hat{I}_{j,y}(t) \rangle$), respectively. These DO observables are normalized with respect to the theoretical maximum amount of DO capable of existing on the entire ^{1}H spin system. In this case, *ca*. 35% of the theoretical maximum amount of DO resides on the ^{1}Hs of the abundant pool after demagnetization (blue curve), whereas the DO of the *X*-coupled ^{1}H pool, spans roughly 7% of the theoretical total (green curve). The purple curve shows the dynamics of the heteronuclear dipolar order state (*i.e.*, $\langle \widehat{IS}(t) \rangle = \langle \hat{I}_{1,z}(t)\hat{S}_z(t) \rangle$) over the course of ^{1}H ADRF, which does not become populated as indicated by $\langle \widehat{IS}(\tau_{\text{ADRF}}) \rangle = 0$.

The total spin density matrix after ^{1}H ADRF was then propagated under the effects of a single *X*-channel pulse (*i.e.*, a single CP contact only) applied along the $+x$-axis. These dynamics are investigated in **Figure S11B** and **S11C**. The data points presented in **Figure S11B** are the expectation values of the abundant pool's DO (blue points, $\langle \hat{F}_{\text{DO}}(\tau_{\text{X}}) \rangle$) and the dilute pool's x-component of spin polarization (red points, $\langle \hat{S}_{\text{x}}(\tau_{\text{X}}) \rangle$) after τ_{X} = 10 ms of *X*-channel RF, both plotted as a function of the *X* RF field strength, $\nu_{1\text{X}}$. As the value of $\nu_{1\text{X}}$ increases, the amount of spin polarization transferred to the dilute pool also increases (as indicated by the red *y*-axis, which is normalized with respect to a fully *z*-polarized dilute pool); interestingly, the total amount of ^{1}H DO residing on the *X*-decoupled abundant pool decreases commensurately. For $\nu_{1\text{X}} >$ *ca*. 55 kHz (which is roughly the ^{1}H linewidth), the amount of transferred $\langle \hat{S}_{\text{x}}(\tau_{\text{X}}) \rangle$ polarization saturates at

about 16%, which is mirrored in $\langle\hat{F}_{\mathrm{DO}}(\tau_{\mathrm{X}})\rangle$ whereby *ca*. 10% of the total abundant ^{1}H DO is depleted. Beyond this RF field strength, there are no dipolar couplings that can facilitate the $\hat{F}_{\mathrm{DO}} \rightarrow \hat{S}_{\mathrm{x}}$ transfer of polarization, and therefore the reduction of the abundant pool's DO happens in response to CP-driven changes to $\hat{I}_{\mathrm{DO}}$. The data in **Figure S11C** show the dynamics of the ^{1}H DO for both the *X*-coupled ^{1}H pool ($\hat{I}_{\mathrm{DO}}(t)$, green curve) and the *X*-decoupled abundant pool ($\hat{F}_{\mathrm{DO}}(t)$, blue curve), over a spin diffusion time τ_{SD}, after an ideal *X* CP transfer assumed to leave the former completely depleted (*i.e.*, $\langle\hat{I}_{\mathrm{DO}}(t=0)\rangle = 0$). Here, we see the two DO curves approaching one another and rapidly settling at $t > 10$ µs, which indicates that these initial out-of-equilibrium dipolar-ordered states reach a new quasi-equilibrium state in the timescale of the inverse of the ^{1}H

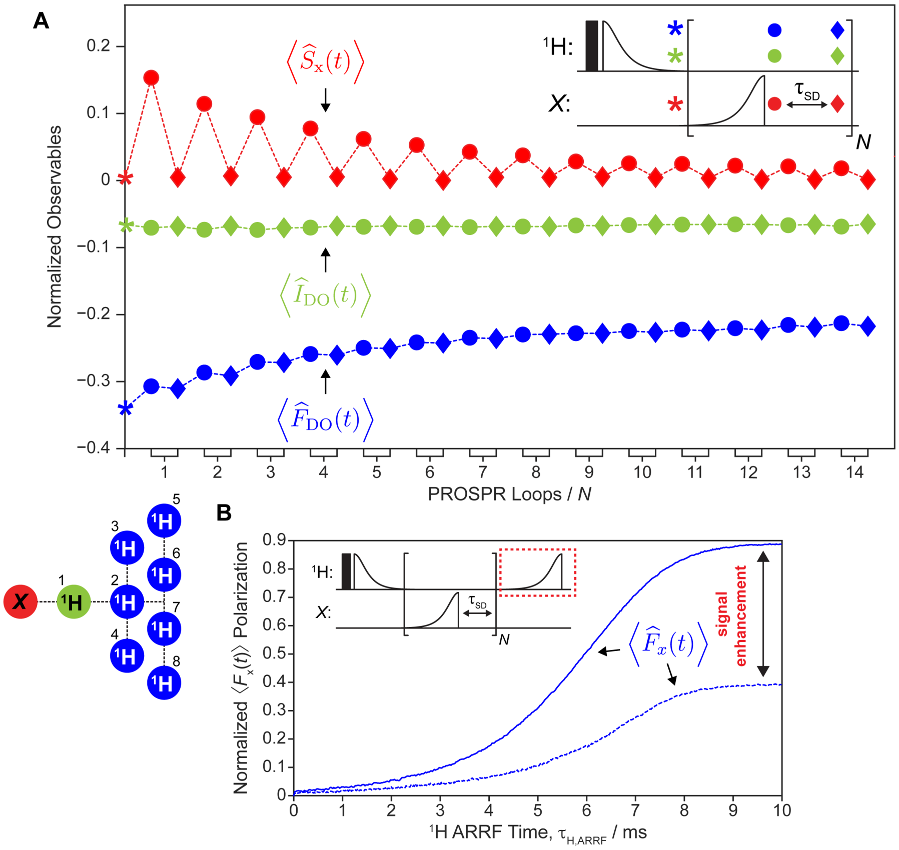

Figure S12. Hilbert-space numerical simulations of dipolar- and Zeeman-order (indicated in each panel) for the same spin system described in **Figure S11** over the course of (**A**) PROSPR with $N = 14$ loops and $\tau_{\mathrm{SD}} = 100$ ms and (**B**) ^{1}H ARRF after $N = 20$ PROSPR loops and $\tau_{\mathrm{SD}} = 100$ ms in the presence (dotted blue curve) and absence (solid blue curve) of *X* RF. In (**A**), the different colours refer to the different observables (indicated in the figure) and the different shapes indicate the time points at which each observable is sampled, both of which are schematically indicated on the PROSPR pulse sequence in the inset. Asterisks denote the observables immediately after ^{1}H ADRF, whereas the circles and diamonds denote the observables immediately after *X* CP and the spin diffusion delay ($\tau_{\mathrm{SD}} = 100$ ms in this case), respectively. All other relevant details are provided in the text.

linewidth (**Figure S11C**, inset). This is in complete analogy to spin diffusion of longitudinal polarization between Zeeman-ordered eigenstates.

Based on these dynamics during and after a single *X* dipole-based CP transfer, **Figure S12** examines the effect of looping these two processes. **Figure S12A,** based on the same spin network as **Figure S11**, shows the expectation values of the abundant ^{1}H pool's DO (blue, $\langle\hat{F}_{\text{DO}}\rangle$), the *X*-coupled ^{1}H pool's DO (green, $\langle\hat{I}_{\text{DO}}\rangle$), and the x-component of ^{195}Pt spin polarization (red, $\langle\hat{S}_{\text{x}}\rangle$) at three distinct time points (indicated by the different geometrical shapes) throughout the PROSPR pulse sequence (schematically shown in the inset) as a function of the number of loops, *N*. After the first *X* contact (going from the asterisks to the adjacent circles), the ^{195}Pt pool is polarized to a value of $\langle\hat{S}_{\text{x}}(\tau_{\text{X}})\rangle = ca.$ 16% (same as in **Figure S11B**), $\langle\hat{I}_{\text{DO}}(\tau_{\text{X}})\rangle$) remains largely unchanged, and $\langle\hat{F}_{\text{DO}}(\tau_{\text{X}})\rangle$ is partially saturated. Over the course of τ_{SD} (going from the first circle to its adjacent diamond), it is evident that the ^{195}Pt undergoes dephasing due to ^{1}H-*X* dipolar coupling ($\langle\hat{S}_{\text{x}}(\tau_{\text{SD}})\rangle = 0$) and the DO for both ^{1}H pools does not change significantly. This behaviour is observed for subsequent CP contacts (going from diamonds to circles), in which the greatest saturation of $\langle\hat{F}_{\text{DO}}\rangle$ occurs over the course of *X* RF and not over the τ_{SD} interval. These numerical results are a consequence of the finite spin system, the absence of relaxation, and the fact that the diffusion of dipolar order can be very rapid (as shown in **Figure S11C**, and demonstrated in the literature[22]), which means that the quasi-equilibrium state characterizing $\hat{I}_{\text{DO}} + \hat{F}_{\text{DO}}$ is rapidly established over the course of the *X* RF pulse, and any additional τ_{SD} time does not improve the repolarization of $\hat{I}_{\text{DO}}$. It is worth noting that the amount of $\langle\hat{S}_{\text{x}}(\tau_{\text{X}})\rangle$ transferred with each contact depends almost entirely on the amount of $\langle\hat{F}_{\text{DO}}\rangle$, and since the latter decreases with each subsequent loop (due to $\hat{F}_{\text{DO}} \rightarrow \hat{I}_{\text{DO}}$ spin diffusion), the amount of transferred polarization also decreases with looping. At this point, the spin density matrix is propagated under the effects of ^{1}H ARRF applied along the $+x$ axis (**Figure S12B,** $\tau_{\text{H,ARRF}} = 10$ ms), which serves to remagnetize the ^{1}H DO of both the *X*-coupled and the *X*-decoupled ^{1}Hs into detectable transverse spin polarization. The evolution of the *X*-decoupled abundant pool's remagnetized transverse spin polarization $\langle\hat{F}_{\text{x}}(t)\rangle$ is shown with the dotted blue curve, which reaches a remagnetization of *ca.* 40%. This effectively confirms PROSPR's two-step saturation-based mechanism for facilitating the transfer of spin polarization/saturation between two dipolar-decoupled pools. To further demonstrate this, the solid blue curve in **Figure S12B** shows $\langle\hat{F}_{\text{x}}(t)\rangle$ over the course of ^{1}H ARRF after the exact

same number of N and τ_{SD}, in the absence of X RF. In this case, $\langle \hat{F}_x(\tau_X) \rangle$ = *ca*. 90 %, which of course is about twice as larger than $\langle \hat{F}_x(\tau_X) \rangle$ with the X channel turned on (solid *vs*. dotted curves). These data also demonstrate the maximum theoretical enhancement, which is given by half of the total number of spins occupying the abundant pool.

Supplementary Note 4: PROSPR as a Two-Site Exchange Using the Bloch-McConnell Formalism: Basic Features about PROSPR's Point-Spread Function

This supplement models PROSPR NMR using CEST-like numerical simulations[23,24] based on a modified Bloch-McConnell formalism,[25,26] which only considers the second step of PROSPR's multi-step mechanism, and treats the spin diffusion of ^{1}H-^{1}H dipolar order as a simple two-site exchange between the X-coupled ^{1}H (dilute) spin pool, and the abundant ^{1}Hs. **Table S14** summarizes the similarities between CEST and PROSPR, which facilitates an understanding of how semi-classical CEST-like simulations can model some of the more complicated PROSPR spin dynamics described in **Supplement 3**. Specifically, we treat the dipolar order reservoirs as longitudinal magnetizations that exchange between two unequally-populated and individually-addressable pools: one is identified as the dilute, strongly X-coupled ^{1}H spins (pool A), and the other as the abundant X-decoupled ^{1}H spins (pool B). A continuous-wave (CW) RF pulse is applied on the A pool of this system that mimics the depletion brought about by heteronuclear ^{1}H(A)→X CP. This RF is assumed to have an effective field ω_1, reflecting the strength of the heteronuclear CP transfer rate, and an offset dependence $\left(\omega_{\mathrm{shift}}^{X} - \omega_{\mathrm{RF}}\right)$, reflecting the position at which the ω_1 field performing the CP is applied within the X-spin powder pattern. The fact that the A spins are dipolar-coupled to X, while the B pool is not, is also reflected by a heteronuclear dipolar broadening ω_{XA} that has no B-site counterpart. Modified Bloch-McConnell equations reflecting all these considerations, are

$$\frac{dM_{\mathrm{x}}^{\mathrm{A}}}{dt} = -\left(\omega_{shift}^{X} - \omega_{\mathrm{RF}}\right)M_{\mathrm{y}}^{\mathrm{A}} - k_{2,\mathrm{A}}M_{\mathrm{x}}^{\mathrm{A}} + k_{\mathrm{ex,B}}M_{\mathrm{x}}^{\mathrm{B}}$$

$$\frac{dM_{\mathrm{y}}^{\mathrm{A}}}{dt} = \left(\omega_{shift}^{X} - \omega_{\mathrm{RF}}\right)M_{\mathrm{x}}^{\mathrm{A}} - k_{2,\mathrm{A}}M_{\mathrm{y}}^{\mathrm{A}} + k_{\mathrm{ex,B}}M_{\mathrm{y}}^{\mathrm{B}} - \omega_1 M_{\mathrm{z}}^{\mathrm{A}}$$

$$\frac{dM_{\mathrm{z}}^{\mathrm{A}}}{dt} = -\frac{M_0^{\mathrm{A}}}{T_1^{\mathrm{A}}} - k_{1,\mathrm{A}}M_{\mathrm{z}}^{\mathrm{A}} + k_{\mathrm{ex,B}}M_{\mathrm{z}}^{\mathrm{B}} + \omega_1 M_{\mathrm{y}}^{\mathrm{A}}$$

$$\frac{dM_x^B}{dt} = -k_{2,B}M_x^B + k_{ex,A}M_x^A$$

$$\frac{dM_y^B}{dt} = -k_{2,B}M_y^B + k_{ex,A}M_y^A$$

$$\frac{dM_z^B}{dt} = -\frac{M_0^B}{T_1^B} - k_{1,B}M_z^B + k_{ex,A}M_z^B$$

where

$$k_{1,A/B} = \frac{1}{T_1^{A/B}} + k_{ex,A/B}$$

$$k_{2,A/B} = \frac{1}{T_2^{A/B}} + k_{ex,A/B} + \omega_{XA}$$

and $\frac{k_{ex,A}}{k_{ex,B}} = \frac{M_0^B}{M_0^A}$ reflect the different populations of the A and B pools.

Table S14. Comparing physics and common features between *in vivo* CEST and PROSPR

CEST		PROSPR
Pool A – labile ^{1}Hs	⟷	Dilute, X-coupled ^{1}H spins
Pool B – water	⟷	Abundant, *X*-decoupled ^{1}Hs
Longitudinal magnetization	⟷	^{1}H-^{1}H dipolar order
Two-site chemical exchange between pool A and pool B	⟷	Spin diffusion of dipolar order between *X*-coupled ^{1}Hs and *X*-decoupled ^{1}Hs
Chemical exchange rate	⟷	Spin diffusion rate
Selective RF saturation of pool A's longitudinal magnetization	⟷	^{1}H-*X* ADRF-CP-based saturation of *X*-coupled ^{1}H dipolar order
The excitation/saturation bandwidth of the CW RF	⟷	CP bandwidth of the *X*-channel RF
Saturation time	⟷	Total spin diffusion time, $N\times\tau_{SD}$
Pool B's longitudinal relaxation time, T_1(B)	⟷	Lifetime of *X*-decoupled ^{1}H-^{1}H dipolar order, T_{1D}(^{1}H)
Pool B's chemical shift $\Delta\nu$(B)	⟷	*X*-nucleus anisotropy (chemical shift, quadrupolar broadening) plus *X*-^{1}H dipolar coupling
Ratio of populations between pool A and pool B, $M_{0,A}/M_{0,B}$	⟷	Ratio of populations between the dilute *X* pool and the abundant *X*-decoupled ^{1}H pool

Figure S13 shows PROSPR numerical simulations based on solutions to modified Bloch-McConnell equations for two site exchange, performed with parameters resembling their experimental counterparts. For instance, the ratio between the initial magnetization of pool A to pool B, proportional to the population of each pool, was set based on the % of ^{195}Pt (considering its natural abundance of 33%) to ^{1}Hs in cisplatin: $M_{0,A}/M_{0,B} = 0.0017$. A T_1 for pool B of $T_1(B) = 13$ s was used; the exchange rate was set based on the experimental ^{1}H NMR spectral linewidth of cisplatin (*i.e.*, the average proton homonuclear dipolar coupling constant, $k_{BA} = 20$ kHz) and the reverse exchange rate ($k_{AB} = 34$ Hz) was set based on mass balance.[23] The total spin diffusion time, $N \times \tau_{SD}$, which is simulated by the pulse length of the CW pulse is based on an experimental N/τ_{SD} combination of $N = 100$ and $\tau_{SD} = 250$ ms. All other relevant simulation parameters are contained in the figure caption. **Figure S13A** shows the saturation of the B pool's abundant ^{1}H magnetization as a function of both the total spin diffusion time and the ^{195}Pt RF field strength, which is resonant with the most intense part of the ^{195}Pt pattern (−440 kHz). These data suggest that at a given ^{1}H(A)$\rightarrow^{195}$Pt CP efficiency, longer spin diffusion times lead to larger depletion of pool B's abundant ^{1}H magnetization; the same is observed for stronger CP strengths at a given spin diffusion time. This is in accordance with CEST-based considerations. **Figure S13B** explores the effects on the ^{195}Pt PROSPR *z* spectra for different CP field strengths. These spectra show that as the field strengths increase, a larger proportion of abundant ^{1}H magnetization is saturated, but the rasterization quality of the ^{195}Pt pattern on the abundant ^{1}Hs is reduced: a significant broadening of the low- and high-frequency discontinuities is then noticed. This effect was referenced in the main text (*e.g.*, **Figure 3**), and is also akin to what is observed in CEST.[29] High RF thus lowers the nominal spectral resolution of the *X* pattern detected on the abundant ^{1}Hs, which means using RF offset step sizes smaller than the RF field (in kHz) will not improve the effective *X* pattern resolution. In **Figure S13C**, the ^{195}Pt PROSPR *z* spectra were simulated at three distinct spin diffusion times, indicated by the three different colours and correspond to the arrows in the inset. Broadening of the spectral discontinuities is not observed and the relative intensity across the entire pattern is well preserved at short saturation times (red), intermediate saturation times (blue), and at saturation times well into the steady state (magenta), where saturation is maximized. Lastly, **Figure S13D** confirms that the % of abundant ^{1}Hs that are saturated increases when their relaxation time increases and when the population difference between the exchanging pools also increases.

This demonstrates that PROSPR can lead to dramatic signal enhancements when targeting severely dilute nuclei in systems that are have long $T_{1D}(^1H)$ relaxation-time constants.

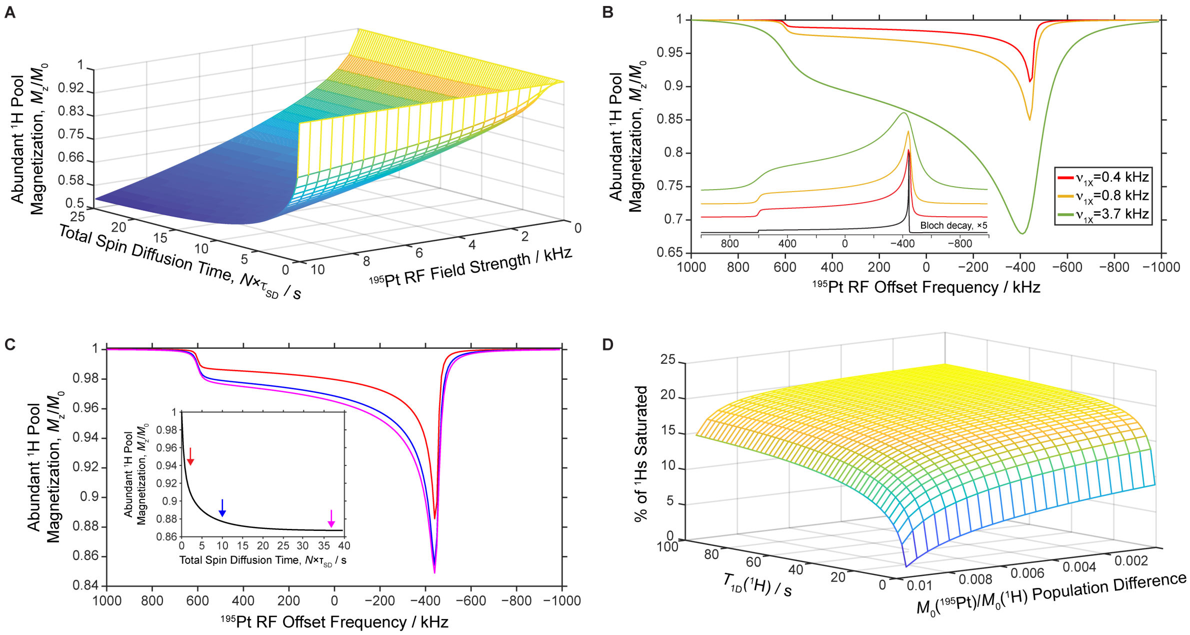

Figure S13. MATLAB-based PROSPR numerical simulations based on the Bloch-McConnell formalism for two-site chemical exchange. (**A**) shows the saturation of abundant ^1H magnetization measured selectively for pool B as a function of both the total spin diffusion time and the ^{195}Pt RF field strength. (**B**) and (**C**) show the ^{195}Pt PROSPR z spectra simulated at different ^{195}Pt RF field strengths (**B**, indicated in the legend) and at different spin diffusion times (**C**, indicated by the coloured arrows), respectively. The inset in (**B**) shows the S_0–S PROSPR spectra plotted against an idealized ^{195}Pt Bloch decay spectrum (black, scaled vertically 5×) that was simulated with the same initial magnetization, *i.e.*, $M_0(^{195}Pt) = 0.0017$ as pool A. The inset in (**C**) was simulated with the ^{195}Pt RF offset frequency resonant with the low-frequency discontinuity (−440 kHz). (**D**) shows the % saturation of pool B's ^1Hs as a function of their dipolar-order relaxation time and the population difference between the two pools. Unless stated otherwise, a total spin diffusion time of 25 s was simulated along with a ^{195}Pt RF field strength of $\nu_{1X} = 0.8$ kHz. In all cases, an axially symmetric ^{195}Pt CS tensor was simulated, having a span $\Omega = -700$ kHz and an isotropic shift of $\delta_{iso} = -100$ kHz (according to the Herzfeld-Berger convention). The average over the powder was calculated using 1200 crystallites uniformly distributed from 0 to π and $\mathbf{B}_0 = 14.1$ T.

Supplementary Note 5: Comparing the Sensitivity and Spectral Resolution Afforded by ADRF-CP, PROSPR, and CP/MAS for the ^{15}N NMR Spectroscopy of natural abundance Ammonium Sulphate

This Supplement provides a comparison between the sensitivity and spectral resolution afforded by PROSPR (top, **Figure S14**), ADRF-CP (middle, **Figure S14**), and conventional CP/MAS (recorded at a spinning speed of 5 kHz, bottom **Figure S14**) using the natural abundance ^{15}N signal of ammonium sulphate as a representative example of a non-quadrupolar, non-wideline

acquisition. It is clear from these data, that under conditions of optimized heteronuclear decoupling (lasting over an acquisition time totalling 100 ms) and 5 kHz magic-angle spinning, the two distinct ^{15}N sites can be reasonably resolved on the basis of chemical shift differences. However, despite its lack of decoupling- and MAS-driven line narrowings, the sensitivity advantages afforded by PROSPR over both CP/MAS and static ADRF-CP, are still evident.

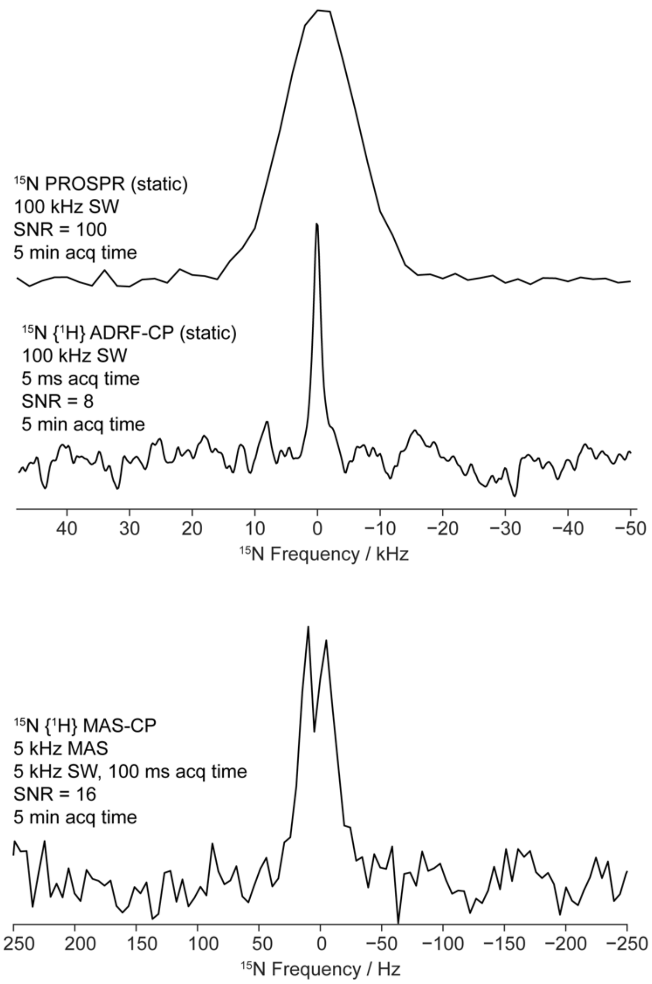

Figure S14. Experimental ^{15}N spectra collected at 14.1T for a sample of ammonium sulphate using the PROSPR (top), ADRF-CP (middle), and CP/MAS (bottom) pulse sequences. Relevant acquisition parameters are listed in the figure.

References:

(1) Stephenson, D.; Smith, J. A. S. *Zeitschrift für Naturforsch. A* **2000**, *55*, 37–40.

(2) Lucier, B. E. G.; Reidel, A. R.; Schurko, R. W. *Can. J. Chem.* **2011**, *89*, 919–937.

(3) Hartmann, S. R.; Hahn, E. L. *Phys. Rev.* **1962**, *128*, 2042–2053.

(4) Ramanathan, C.; Ackerman, J. L. *J. Magn. Reson.* **1997**, *127*, 26–35.

(5) Harris, K. J.; Lupulescu, A.; Lucier, B. E. G.; Frydman, L.; Schurko, R. W. *J. Magn. Reson.* **2012**, *224*, 38–47.

(6) O’Dell, L. A.; Schurko, R. W. *Chem. Phys. Lett.* **2008**, *464*, 97–102.

(7) MacGregor, A. W.; O’Dell, L. A.; Schurko, R. W. *J. Magn. Reson.* **2011**, *208*, 103–113.

(8) Veinberg, S. L.; Lindquist, A. W.; Jaroszewicz, M. J.; Schurko, R. W. *Solid State Nucl. Magn. Reson.* **2017**, *84*, 45–58.

(9) Massiot, D.; Farnan, I.; Gautier, N.; Trumeau, D.; Trokiner, A.; Coutures, J. P. *Solid State Nucl. Magn. Reson.* **1995**, *4*, 241–248.

(10) Medek, A.; Frydman, V.; Frydman, L. *J. Phys. Chem. A* **1999**, *103*, 4830–4835.

(11) Dell, L. A. O.; Rossini, A. J.; Schurko, R. W. *Chem. Phys. Lett.* **2009**, *468*, 330–335.

(12) Schurko, R. W. *Acc. Chem. Res.* **2013**, *46*, 1985–1995.

(13) Grad, J.; Bryant, R. G. *J. Magn. Reson.* **1990**, *90*, 1–8.

(14) Altenhof, A.; Jaroszewicz, M.; Harris, K.; Schurko, R. *J. Chem. Phys.* **2021**, *154*, 34202.

(15) Raya, J.; Perrone, B.; Hirschinger, J. *J. Magn. Reson.* **2013**, *227*, 93–102.

(16) Johnson, R. L.; Schmidt-Rohr, K. *J. Magn. Reson.* **2014**, *239*, 44–49.

(17) Saïdi, F.; Taulelle, F.; Martineau, C. *J. Pharm. Sci.* **2016**, *105*, 2397–2401.

(18) Raya, J.; Hirschinger, J. *J. Magn. Reson.* **2017**, *281*, 253–271.

(19) Lupulescu, A.; Frydman, L. *J. Chem. Phys.* **2011**, *135*, 134.

(20) Haeberlen, U. *High Resolution NMR in Solids : Selective Averaging*; Academic Press: New York, 1976.

(21) Baum, J.; Munowitz, M.; Garroway, A. N.; Pines, A. *J. Chem. Phys.* **1985**, *83*, 2015–2025.

(22) Dementyev, A. E.; Cory, D. G.; Ramanathan, C. *Phys. Rev. B - Condens. Matter Mater. Phys.* **2008**, *77*, 1–5.

(23) Woessner, D. E.; Zhang, S.; Merritt, M. E.; Sherry, A. D. *Magn. Reson. Med.* **2005**, *53*, 790–799.

(24) Helgstrand, M.; Hard, T.; Allard, P. *J. Biomol. NMR* **2000**, *18*, 49–63.

(25) McConnell, H. M. *J. Chem. Phys.* **1958**, *28*, 430–431.

(26) Woessner, D. E. *J. Chem. Phys.* **1961**, *35*, 41–48.

(27) Zaremba, S. . *Ann. di Mat. Pura ed Appl.* **1966**, *4:73*, 293.

(28) Conroy, H. *J. Chem. Phys.* **1967**, *47*, 5307–5318.
